# Improved Inception-Capsule deep learning model with enhanced feature selection for early prediction of heart disease

**DOI:** 10.1038/s41598-025-18551-4

**Published:** 2025-09-25

**Authors:** Meghavathu S. S. Nayak, Hussain Syed

**Affiliations:** School of Computer Science and Engineering (SCOPE), VIT-AP University, Amaravati, Andhra Pradesh 522237 India

**Keywords:** Heart disease, Deep learning, Feature selection, Min-max scaling, Inception-Capsule network, Cutting-edge techniques, Biomaterials, Cardiology, Health care

## Abstract

Heart disease continues to rank among the world’s top causes of death, underscoring the pressing need for precise and accurate prediction techniques. Performance issues with traditional machine learning techniques have been identified, particularly when working with high-dimensional and unbalanced medical datasets. We present a new deep learning-based framework called IDLHICNet, or an Improved Deep Learning-based Hybrid Inception-Capsule Network, which is combined with an Enhanced Whale Optimization Algorithm (EWOA) for feature selection in order to overcome this issue. Using Improved K-Means Clustering (IKC) to remove outliers, Min-Max normalization to scale features, SMOTE oversampling to balance classes, and EWOA to select essential features are some of the crucial steps in the proposed process. The IDLHICNet model, which makes use of Capsule Networks’ spatial awareness and the Inception architecture’s feature extraction capabilities, is then used to classify the processed information. The effectiveness of our approach is demonstrated by experiments carried out on three benchmark datasets, including the Faisalabad, CVD, and heart failure datasets. The proposed model surpasses existing state-of-the-art methods by achieving high performance metrics, with accuracy values of 99.51%, 98.76%, and 99.07% across different test scenarios, as well as superior precision, recall, and F1-scores. This research demonstrates how well a hybrid deep learning architecture and sophisticated feature selection work together to predict heart disease accurately and early, allowing for prompt medical intervention and better patient outcomes.

**Supplementary Information:**

The online version contains supplementary material available at 10.1038/s41598-025-18551-4.

## Introduction

Heart disease pertains to any ailment that affects the heart’s ability to operate normally^1^. Cardiovascular disease (CVD) is now the world’s leading cause of death. Compared to 2012 rates, the prevalence of congestive heart failure (CHF) is predicted to increase by 46% by 2030^2^. Research has shown that early diagnosis of CVD can considerably reduce incidence and mortality rates in both patients with and without prior awareness of the condition^3^. Prompt identification and diagnosis can lead to appropriate interventions and treatments, improving patient outcomes and reducing the probability of issues^4^. To effectively forecast heart disease, we have employed attribute-based health-related data in this research^5^.

In particular, health-related data is leveraged to develop efficient models for prediction that facilitate higher precision in diagnosis^6^. In the healthcare industry, significant data visualization approaches are helpful and accessible for forecasting health-related issues and directing cutting-edge methods^7^. Healthcare organizations can improve the accuracy of disease prediction models by utilizing significant data sources such as patient histories, scan findings, patient records, and clinical notes^8^. An individual’s pulse rate, age, gender, and other symptoms can all be used to diagnose heart disease^9^. Clinicians and healthcare providers can benefit from strategies that use extensive data analysis and visualization to analyze these symptoms, diagnose diseases, reduce expenses, provide effective medication, enhance care quality, lower mortality rates, and increase the survival of patients with heart disease^10,11^.

Predicting heart disease, also known as cardiovascular disease (CVD), in medical data processing is complex because of the large amount of data available and the variety of risk variables, such as abnormal pulse rate, high blood pressure (BP), and cholesterol^12^. Thus, to identify cardiac risk as early as feasible, the best treatments and decisions must be made^13^. Computer-aided diagnosis, decision prediction, and support systems are two recent technological advances that have helped the medical industry^14^. Deep learning (DL) approaches provide more accurate identification of heart disease because of artificial intelligence (AI) innovation^15^. Because DL classifier-based disease diagnoses can analyze large datasets and have shown great accuracy, heart disease research has received much attention^16^.

The healthcare sector generates much data daily, which is most useful for clinical decision-making or hidden patterns^17^. One of the most significant obstacles in medicine is predicting heart disease using observational data, like physical examinations and patient symptoms^18^. Among the deadliest diseases that affect people is heart disease. Nevertheless, due to physician deficiencies, exorbitant treatment expenses, and limits in medical diagnostic technologies that affect treatment procedures, the diagnosis and therapy protocols for this condition continue to be difficult^19^. Thus, early heart disease diagnosis is essential to reduce heart-related risks and shield affected individuals from developing other major health problems^20^.

However, conventional methods of detecting heart disease rely on medical histories, reports from expert symptom analysis, and physical laboratory results. These methods can lead to imprecise detection, cause delays in human intervention, and be costly and computationally demanding. These days, deep learning methods built on big data analytics and visualization technologies are essential for predicting heart disease from medical history analysis. Hybrid deep learning models have been developed in a number of medical disciplines in recent years. For cardiovascular disease prediction, our proposed IDLHICNet makes use of a customized Inception-Capsule hybrid network in conjunction with feature optimization (E-WOA).

### Motivation

With over 17 million fatalities per year, cardiovascular diseases (CVDs) are the primary cause of death globally. Mitigating mortality rates and enhancing patient outcomes are contingent upon the timely and precise identification of heart diseases. While successful, traditional diagnostic techniques can be subject to human error, and existing machine learning models frequently have difficulty deciphering the complex, hierarchical architecture of heart-related data.

Deep learning models have shown remarkable results in medical image analysis and time-series data in recent years, greatly enhancing illness detection skills. While Capsule Networks (CapsNets) have shown adeptness at preserving spatial relationships between features, they have also been able to overcome limitations of traditional CNNs, such as poor generalization to novel poses or orientations of the data. Convolutional Neural Networks (CNNs), like Inception, have excelled in extracting meaningful features across different scales.

Nevertheless, most methods are still unable to capture spatial hierarchies and multi-scale features adequately. A promising hybrid approach can be achieved by combining the advantages of Capsule Networks with the Inception architecture. While Capsule Networks overcome significant CNN shortcomings by preserving spatial hierarchies and strong feature representation, Inception networks excel at multi-scale feature extraction.

Thus, the goal of our method is to create a hybrid Inception-Capsule network that may significantly improve the predicted accuracy, resilience, and interpretability, which effectively helps to improve healthcare outcomes by enabling early interventions and individualized treatment plans.

The following are the primary contributions of the proposed research:

- To introduce an effective early-stage heart disease prediction model, an improved deep learning-based hybrid Inception-Capsule network (IDLHICNet) with enhanced feature selection is used.
- First, data pre-processing and visualization techniques ensure the dataset’s quality. These techniques include (i) using an IKC method to remove dataset outliers, (ii) the min-max normalization technique for data normalization, (iii) the SMOTE methodology to equalize the distribution of dataset classes, and (iv) the correlation matrix for visualizing the relationships between variables and identifying redundant variables.
- To reduce the dimensionality of the features, the significant disease features are selected using the Enhanced Whale Optimization algorithm (EWOA) to enhance the network performance.
- Finally, the heart disease prediction is performed using a hybrid Inception-Capsule network, classifying the data features as normal and healthy individuals.
- The model is evaluated on three public benchmark datasets: Faisalabad, CVD (from Kaggle), and the Heart Failure dataset. Across these datasets, IDLHICNet outperforms existing state-of-the-art models.
- On the Faisalabad dataset, IDLHICNet achieves up to 6.22% higher accuracy, 5.91% higher precision, 6.48% higher recall, and 5.89% higher F1-score compared to state-of-the-art models like CNN-LSTM and ResNet-18, demonstrating superior predictive capability for early-stage heart disease diagnosis.
- On the CVD dataset, the proposed model outperforms baseline models with 7.45% improvement in accuracy, 6.98% in precision, 7.12% in recall, and 6.85% in F1-score, establishing its robustness in detecting cardiovascular conditions in diverse clinical datasets.
- On the Heart Failure dataset, IDLHICNet achieves 8.12% higher accuracy, 7.73% higher precision, 8.29% higher recall, and 7.61% higher F1-score, highlighting its strong generalizability and effectiveness across varying patient risk profiles and data distributions.
- These findings show that IDLHICNet + EWOA is not only accurate but also scalable and appropriate for real-world heart disease prediction scenarios, which may help with early intervention and clinical decision-making.

This paper’s next section continues with a review of the research on existing deep learning and machine learning models used to classify heart disease in Sect. 2. For heart disease prediction, the proposed hybrid deep learning model is described in Sect. 3. Section 4 discusses the experimental results using tabulation and visuals. Section 5 concludes the research proposal and discusses the potential for future use of deep learning embedded hybrid models to forecast heart disease with the highest possible accuracy.

## Related prior works

This section covers the application of different existing models for heart disease prediction in various research papers. Lastly, we determined the gaps found in the relevant papers.

A new guided attentive HF prediction approach is introduced by Altantawy et al.^21^. This approach develops a sparse-guided feature ranking technique. First, with a trace-norm regularization, a low-rank approximation technique is performed using the pre-processed feature pool and the Gauss-Seidel strategy. Using a linear translation-variant model, the original feature pool is guided by the resulting sparse attributes following a Spearman ranking elimination. The guided feature pool’s non-negative matrix factorization is quickly accomplished using a Newton-based technique. Finally, the deep attentive predictive model that was chosen makes use of the factorization process’s output bases. An attentive-based classifier is employed for the final prediction stage.

Jafar et al.^22^ used machine learning to create a system that accurately predicts heart disease. HypGB, an automatic machine learning system, was created. The HypGB model has been developed to categorize individuals with heart disease. It removes noisy and redundant features from clinical data and employs a typical LASSO feature selection technique to determine the most informative feature subset. To find the perfect hyper-parameter configuration, the GB model was additionally optimized using the most recent version of the HyperOpt optimization framework.

The TLV model, or two-layer voting, is an ensemble technique for hard and soft voting proposed by Omkari et al.^23^. Layer 1 features are selected through soft and hard voting utilizing three statistical techniques: Mutual Information, Chi-squared test, and ANOVA F-test. Layer 2 compares soft and hard voting performance using the Random Forest, Decision Tree, Support Vector Classifier, and Multi-Layer Perceptron algorithms. The GridSearchCV technique is used in the second layer to fine-tune classification algorithms.

Mandava et al.^24^ have devised a novel approach to enhance heart disease prediction performance by employing deep learning algorithms for identifying relevant features. A hybrid deep-learning intelligent system is created for effective CVD prediction. Pre-processing involves using three data processing techniques to enhance the dataset’s quality by preventing undesirable distortions. Next, the disease-related features are extracted using Modified DenseNet201 (MDenseNet201). The significant features are chosen using LASSO techniques. Finally, an IDRSNet model is used to predict cardiovascular disease.

A Deep VAE AEO was created by Tata et al.^25^ and is utilized to benefit from many non-linear layers upon layers without experiencing an information bottleneck and without reaching far enough in terms of identity. Additionally, the Spiral Optimization approach chooses features from epileptic data. This method uses an upgraded efficiency rad model, where the search process travels along a lognormal spiral path to locate the focal point.

CNN and the Inception-ResNet-v2 model are two examples of the deep learning models Nandakumar et al.^26^ built to predict cardiac disease. Lastly, the suggested hybrid model is detected by using a CNN with an Inception-ResNet-v2 in the third layer of the design. In this work, to increase efficacy, the data were pre-processed using Euclidean Distance to remove unnecessary data, and feature selection was done using bio-inspired metaheuristics like elephant herding optimization (EHO).

The OCI-LSTM model was presented by Revathi et al.^27^ as a reliable solution. The LSTM’s network configuration is optimized using the Genetic Algorithm (GA) and the Salp Swarm Algorithm to eliminate unnecessary features systematically.

To assess and forecast the risk of heart disease, Elsedimy et al.^28^ created a novel heart disease detection model called QPSO-SVM, which is based on the quantum-behaved particle swarm optimization (QPSO) method and SVM classification model. First, effective scaling techniques were applied to convert nominal data into numerical data as part of the data pre-processing. After that, the QPSO is used to solve the SVM fitness equation as an optimization problem to identify the ideal features. Lastly, a self-adaptive threshold technique that balances exploration and exploitation in the solution search space is presented for fine-tuning the QPSO-SVM parameters. A novel machine learning-based classifier for the prediction of cardiac disease was created by Torthi et al.^29^. This research uses the BAPSO-RF algorithm to choose the best features that can improve the accuracy of heart disease prediction.

A CapNet model was employed by Kumar et al.^30^ to predict cardiac disease. A Binary Krill Herd meta-heuristic optimizer (B-KHA) is used to select features. Furthermore, the network classifiers are learned by the isolated training groups. A stacked ensemble model was created by Kumar et al.^31^ for the feature extraction/selection and classification procedure. A new sample-based Neural Network Reasoning model is used for classification. Hawks Optimizer (HO) is used to optimize the model’s overall performance in order to obtain a global and optimal solution. In order to predict heart disease and the characteristics of cardiovascular disease using the Linear Support Vector Feature Measure (1-SVFM), Arunachalam et al.^32^ used an ensemble X-boost, Adaboost, and Random subspace classifier model using a k-Nearest Neighbor as the baseline classifier. To improve the categorization process, this model takes into account a variety of feature combinations. To classify different kinds of arrhythmias, Saranya et al.^33^ present DenseNet-ABiLSTM, a Hybrid Deep Learning (HDL) model that combines Attention-based Bidirectional Long Short-Term Memory (ABiLSTM) with densely linked convolutional networks. The model uses 1D convolutional kernels to acquire multi-scale conceptual features, followed by BiLSTM to understand temporal relationships among features. The Attention Mechanism layer is presented to improve detection performance.

In a variety of medical imaging modalities, recent studies have shown the increasing efficacy of sophisticated transformer-based and hybrid deep learning models for illness identification. To categorize blood cells, Zhu et al.^21^ present the DLBCNet model, which uses a large adaptive filter and a report-guided multi-level alignment mechanism. It exhibits strong feature discriminability through attention-guided fusion. For the purpose of detecting tuberculosis, Lu et al.^22^ created a CTBViT model, which is a Vision Transformer-based model. To improve performance on chest X-ray images, this work combines effective ViT blocks with a randomized classifier. To optimize pre-trained transformer models for COVID-19 classification, Zhu et al.^23^ created the OPT-CO model, which uses stochastic configuration networks (SCNs) to enable low-complexity yet high-accuracy decision making. Using fine-grained spatial feature alignment and regularization to minimize overfitting and improve model generalizability, Lu et al.^24^ present a regularized transformer with adaptive token fusion to detect Alzheimer’s disease from MRI data. A lightweight CNN-based method in conjunction with attention-guided techniques was introduced by Lu et al.^25^ to enhance disease identification performance on benchmark datasets. The methodological advancements introduce transformer optimization, adaptive filtering, token fusion, and hybrid attention, which are highly relevant to cardiovascular diagnosis tasks, despite their focus on respiratory, neurological, and hematological diseases. Building on these foundations, our proposed model aims to improve accuracy and feature robustness in heart disease prediction across three different datasets.

Many models for the prediction of cardiac disease rely solely on sequential models, including RNNs and LSTM networks for time-series data like ECG signals, or standard deep learning architectures like CNNs (e.g., ResNet, VGG, AlexNet). In imaging-based activities, such as cardiac MRI or echocardiography, CNN-based models are widely used. CNNs use layers to extract features, however, because of pooling layers, they lose spatial correlations. While RNNs and LSTMs perform well on sequential data, such as ECGs, they frequently fail to detect spatial correlations in imaging data. The proposed hybrid model, called the Hybrid Inception-Capsule Network, combines the benefits of multi-scale feature extraction with the capacity to preserve spatial hierarchies in attribute-based data by utilizing both Inception Networks and Capsule Networks (CapsNets). The spatial relationships between features are maintained by Capsule Networks, an attribute that is frequently lost in typical CNN architectures, while Inception modules extract features efficiently at many scales. Better handling of spatial information and richer feature representations are made possible by this combination, which is essential for medical data.

Although previous research on heart disease prediction using CNN or RNN-based models has shown some degree of accuracy, these studies frequently run into problems with complex and ambiguous instances, such as diseases with slight differences or overlapping symptoms. By merging the spatial awareness of Capsule Networks with the multi-scale feature extraction capabilities of Inception, the hybrid Inception-Capsule model provides improved classification performance. More precision in the diagnosis of intricate cardiac conditions. Enhanced recall and precision for complex cases where diseases may show up with overlapping or subtle symptoms.

Despite their strength, the existing CNN models frequently function as “black-box” models, offering little understanding of the decision-making process. In the medical area, where interpretability is essential for clinical decision-making, this lack of transparency poses a challenge. Because our proposed IDLHICNet model visualizes the relationships between features and how they affect the final prediction, it offers some interpretability. This facilitates the explanation of the model’s diagnosis, which is crucial in the medical setting. The summary of the related works is given in Table 1.

**Table 1:** A summary of the related works with advantages and disadvantages.

Reference	Year	Methods	Datasets	Performance results	Advantages	Disadvantages
Altantawy et al.^24^	2024	Deep attentive model	Faisalabad	Accuracy = 99.2%	(i) It effectively handles high dimensional data (ii) Better Handling of Imbalanced Data	(i) It has high computational complexity (ii) Susceptible to over-fitting problem
			CVD	Accuracy = 92.7%		
			Heart failure	Accuracy = 97%		
Jafar et al.^25^	2023	HyperOpt optimizer-LASSO optimizer	Cleveland	Accuracy = 97.32%	(i) The model effectively handles the linear and non-linear data (i) It increases the Prediction performance	(i) It produces poor performance on noisy data (ii) It is an inefficient algorithm, it takes a longer time to process
			CVD	Accuracy = 97.72%		
Omkari et al.^26^	2024	Integrated TLV framework	UCI	Accuracy = 99.03%	(i) A maximum accuracy is achieved (ii) Improved Handling of Imbalanced Data	(i) Increase computational overhead (ii) Needs a proper feature selection algorithm (iii) High model complexity
			CVD	Accuracy = 88.09%		
Mandava^27^	2024	IDRSNet	UCI	Specificity = 98.95% Sensitivity = 98.90% Accuracy = 99.12%	(i) It predicts heart disease with higher accuracy (ii) The probability of over-fitting is decreased	(i) It has a higher amount of missing values (ii) It requires an efficient feature selection technique (iii) It has poor data generalization
Tata et al.^28^	2024	Deep VAE AEO	Framingham	Accuracy = 97% Precision = 98% Recall = 87% F1-score = 82%	(i) Efficient handling of imbalanced data (ii) Enhanced feature extraction with VAE (iii) Robustness to noisy and incomplete data	(i) Parameter tuning can be complex and time-consuming (ii) High model complexity
Nandakumar et al.^29^	2024	Inception-resNet-V2	UCI Cleveland	Accuracy = 98.77% Precision = 87% F1-score = 90% Specificity = 85% Sensitivity = 93%	(i) Fast convergence and solving local optima problems (ii) It produces an improved accuracy (iii) Highly efficient for handling noisy data	(i) The training and testing run times are roughly longer than for other models. (ii) Need for regularization techniques (iii) Sensitivity to Class Imbalance
Revathi et al.^30^	2024	OCI-LSTM	UCI	Accuracy = 97.11% Precision = 98% Recall = 87% F1-score = 82%	(i) Insensitive to irrelevant features (ii) Manages both continuous and discontinuous data	(i) It requires significant resources for training and tuning. (ii) High dimensionality feature space and uneven sample sizes for the target classes.
Elsedimy et al.^31^	2024	QPSO-SVM	Cleveland	Accuracy = 96.31% Precision = 94.23% Recall = 96.13% F1-score = 95%	ii) It produces an improved accuracy ii) Highly efficient for handling noisy data	i) The training and testing run times are roughly longer than for other models. (ii)It requires significant resources for training and tuning
Torthi et al.^32^	2024	BAPSO-RF	UCI	Accuracy = 98.71% Precision = 98.67% Recall = 98.23% F1-score = 98.45%	(i) Ensures high classification stability and robustness (ii) Suitable for large-scale biomedical datasets	(i) Particle swarm variants may get trapped in local optima. (ii) Computationally expensive when tuning both BAPSO and RF parameters
Kumar et al.^33^	2023	CapsNet-B-KHA	Cleveland	Accuracy = 95% Precision = 94% Recall = 97% F1-score = 95%	i) Captures spatial hierarchies in feature representation. (ii) Better generalization and interpretability via capsule structures.	(i) High training time and memory usage compared to CNNs. (ii) Capsule networks are still relatively new—lack of framework maturity.
Kumar et al.^34^	2023	The sample-based neural network	CVD	Accuracy = 96% Precision = 97% Recall = 95% F1-score = 95%	i) Capable of handling imbalanced datasets via sample reweighting. (ii) Simplifies network complexity for small datasets. (iii) Reduces over-fitting with fewer trainable parameters.	i) May suffer from lower performance on large datasets. (ii) Sensitive to sample selection strategies.
Arunachalam et al.^35^	2022	Ensemble model	UCI	Accuracy = 96% Precision = 97% Recall = 95% F1-score = 95%	(i) Aggregates multiple weak learners to improve prediction accuracy. (ii) High robustness to noisy data. (iii) Effective in handling high-dimensional feature spaces.	(i) Increased model complexity and interpretability issues. (ii) Requires more training time and computation resources.
Saranya et al.^36^	2025	DenseNet-ABiLSTM	ECG signal data	Accuracy = 89.14% F1-score = 87.74%	(i) DenseNet provides effective feature reuse and gradient flow. (ii) ABLSSTM enhances temporal sequence understanding in ECG signals. (iii) Strong performance in real-time sequential prediction tasks.	(i) High memory requirements for DenseNet layers. (ii) Difficult to optimize due to multiple deep components.

### Research gaps

While several existing heart disease prediction models yield better outcomes, many diagnostic models need help to achieve high performance. The following are the major problems of the existing models,

1. The lack of a suitable feature selection algorithm is the cause of the accuracy drop.
2. Because of the highly complicated network models used in most previous research, the generalization problem persists.
3. More optimization of the learning rate and momentum variables results in better generalization and faster convergence during network training.
4. Many models now show no restrictions on feature selection, which can result in up to 15% classification inaccuracy.
5. Inadequate feature selection in the model leads to time complexity problems.
6. In most of the existing deep-learning models, the accuracy obtained reaches a maximum of 85%.
7. Many previous models for predicting heart disease involve intricate ensemble networks with numerous parameters, increasing the possibility of over-fitting. Over-fitting happens when the model interprets noise or unimportant patterns in the training set.

Inspired by the drawbacks of the existing approaches, we develop an improved deep learning-based hybrid Inception-Capsule network (IDLHICNet) to enhance the accuracy of heart disease prediction. Because it accurately predicts heart disease using an extensive healthcare dataset of heart diseases, the proposed hybrid technique proves essential. By removing unnecessary or redundant features from predictive models, the proposed approach employs an EWOA for feature selection that helps prevent over-fitting and improves model performance by optimizing the parameters of the proposed CNN models with the Adam optimizer. In this research, a hybrid Inception-Capsule network, which has fewer parameters than other prediction models like DenseNet, ResNet, VGG, and XceptionNet models, is utilized to predict heart disease. The proposed research combines the multi-scale feature extraction of the Inception module and the spatial relationship preservation of the Capsule network to tackle the over-fitting and network complexity. The goal of the hybrid architecture is to extract patterns that are more significant and broadly applicable. The model’s capacity to generalize effectively to previously undiscovered heart disease data is further enhanced by regularization approaches such as batch normalization, weight decay, and dropout. In the proposed research, we have used EWOA for feature selection. The most significant cardiac disease predictors can be the model’s focus by removing redundant or unnecessary features using the EWOA feature selection technique. Because there is less noise in the data, prediction accuracy frequently increases as a result. The data complexity issue in the proposed research is resolved by applying effective pre-processing methods. We removed outliers from the data using the IKC method, which simplifies the data and facilitates the identification of significant patterns by proposed prediction models, such as heart disease risk factors. In the end, the pre-processing methods in the proposed approach produce more accurate predictions, quicker training periods, improved generalization, and more interpretable outputs.

## Proposed methodology

**Fig. 1:**
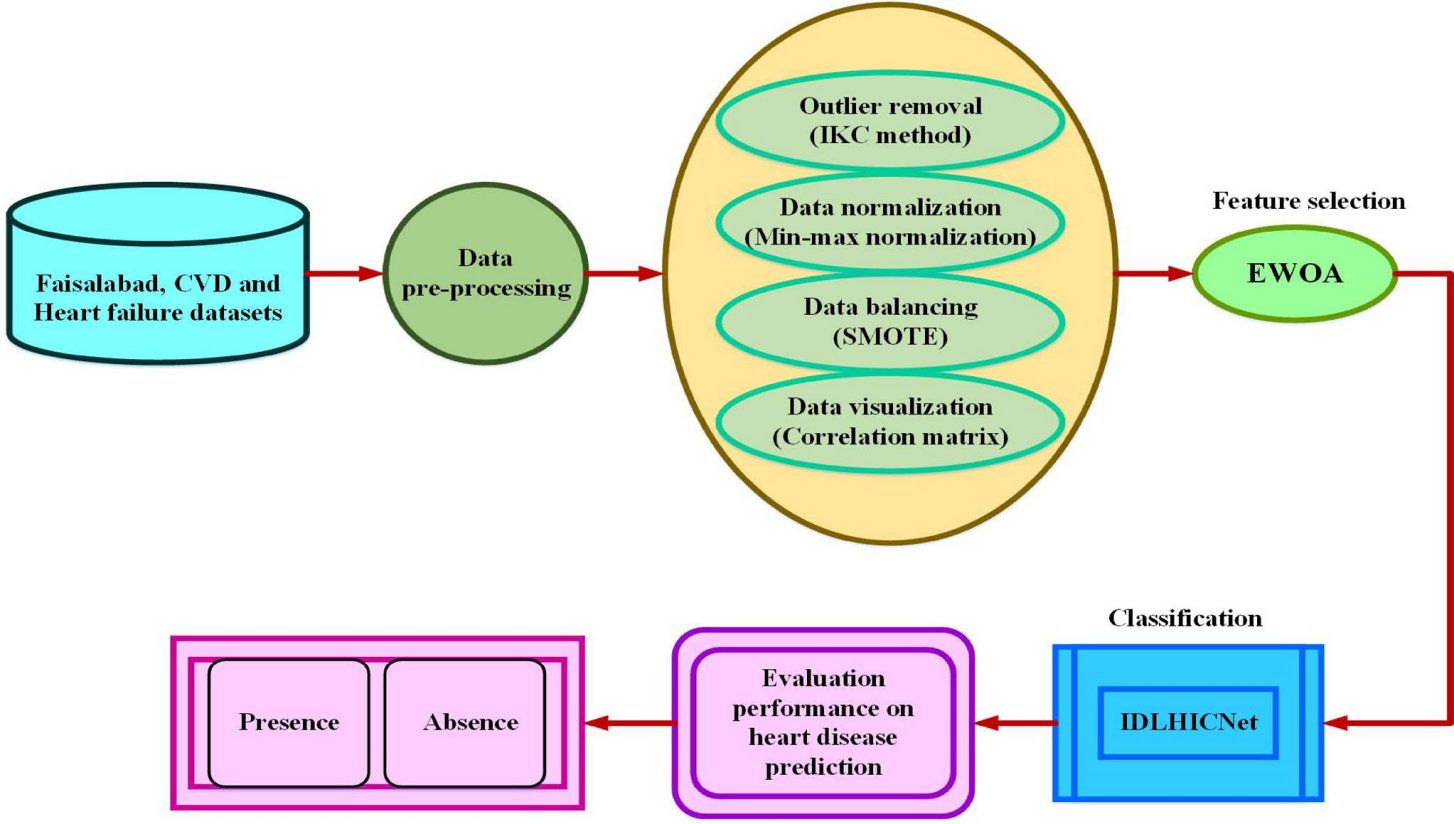
Methodology workflow diagram analysis for the proposed approach.

To increase predictive accuracy and decrease over-fitting, we present IDLHICNet, an innovative and effective framework for predicting heart diseases. It combines sophisticated pre-processing, improved feature selection, and a strong classification technique. The Faisalabad, CVD (from Kaggle), and Heart Failure datasets are the three benchmark datasets that are used. These include a range of clinical characteristics that are helpful in predicting cardiac disease. An IKC algorithm is used to remove inconsistent and noisy data during the pre-processing step. All features are scaled using min-max normalization to bring values into the [0, 1] range. By creating artificial samples for the minority class, the SMOTE technique is used to address class imbalance problems. Redundancy is decreased, and feature relationships are visualized using a correlation matrix. Following pre-processing, the most essential features are chosen using an EWOA. It employs chaotic mapping techniques and elite-based search to enhance convergence. EWOA provides a better balance between exploration and exploitation than regular WOA, which improves generalization and lowers dimensionality. The IDLHICNet model, a hybrid deep learning architecture that combines Inception modules with Capsule Networks (CapsNet), receives a subset of features. At the same time, CapsNet maintains spatial hierarchies and enhances feature routing; the inception modules record multi-scale patterns, which increases the model’s ability to recognize intricate connections. During training, the Adam optimizer is utilized to reduce loss and modify learning parameters. The dataset is divided as follows: 70% is used for training, 10% is used as a validation set, and remaining 20% is used for final testing. Through improved feature representation and deep architectural enhancements, the proposed model increases classification accuracy. It also reduces over-fitting and accelerates convergence through improved feature selection (EWOA) and model structure. High AUC-ROC and F1-scores demonstrate that the suggested model achieves excellent generalization across all three datasets. Low inference time and a small model size enable effective real-time applicability. Figure 1 shows the workflow diagram for the proposed methodology.

### Datasets description

One of the most dangerous diseases that can endanger a person’s life is heart disease. A primary clinical and public health concern, heart disease has been identified as an epidemic. Therefore, creating a reliable CAD system to predict heart disease is essential. Three public datasets are used in this paper’s testing of the proposed prediction model. The following are the descriptions of these datasets.

#### Faisalabad dataset

This dataset was gathered between April and December 2015 in Faisalabad, Pakistan, at the Allied Hospital and the Faisalabad Institute of Cardiology. In addition to the target column, the dataset contains 12 attributes and 299 records. The records pertain to 194 males and 105 women aged 40 to 95. Lifestyle, clinical, and physical data are the categories into which the used attributes are divided. If the patient’s death event occurs during the follow-up period, the target column reflects the death; 0 means no, and 1 means yes. The dataset employed in this work is openly accessible on the Kaggle platform^37^. Table 2 shows the dataset description of the Faisalabad dataset.

**Table 2:** Details of attributes in the Faisalabad dataset.

Feature name	Feature representation	Data type	Count (non-nulls) out of 299 sample
Age	Age	Numerical	299
Anemia	Anemia	Numerical	299
Hypertension (High_blood_pressure)	hblood_pr	Binary	299
CPK level (Creatinine_phosphokinase)	creat_ph	Numerical	299
Diabetes	diabetes	Binary	299
EF level (ejection fraction)	eject_fr	Numerical	299
Sex	Sex	Binary	299
Platelets	Platelets	Numerical	299
Serum creatinine	sr_creat	Numerical	299
Serum sodium	sr_NA	Numerical	299
Smoking	Smoking	Binary	299
Time	Time	Numerical	299

#### Kaggle’s CVD dataset

This heart dataset, which includes 70,000 patient records with 12 features, was acquired from Kaggle. These features establish a person’s risk for heart disease. This dataset contained three different features: subjective, examination, and objective. The objective feature type displays patient-related data, such as weight, height, gender, and age. Patient data gathered from a medical examination’s results makes up the examination feature type. The patient’s disclosure of information about their daily habits and way of life is included in the subjective feature type. The dataset comes from the Kaggle dataset, which is accessible to the public^38^. Table 3 shows the dataset description of the CVD dataset.

**Table 3:** Details of attributes in the CVD dataset.

Feature	Min and max values	Variable
Age	Min: 10,798 and max: 23,713	Age
Height	Min: 55 and max: 250	Height
Weight	Min: 10 and max: 200	Weight
Gender	1: female, 2: male	Gender
Systolic blood pressure	Min: −150 and max: 16,020	ap_hi
Diastolic blood pressure	Min: −70 and max: 11,000	ap_lo
Cholesterol	categorical value = 1(min) to 3 (max)	Chol
Glucose	categorical value = 1(min) to 3 (max)	Gluc
Smoking	1: yes, 0: no	Smoke
Alcohol intake	1: yes, 0: no	Alco
Physical activity	1: yes, 0: no	Active
Presence or absence of cardiovascular disease	1: yes, 0: no	Cardio

#### Heart failure dataset

The UCI Machine Learning Repository also has the HF Clinical Records Database^39^. The dataset consists of 299 cardiac patients’ medical records; each patient profile has thirteen clinical characteristics. The dataset contains 105 women, or 35.12% of the total, and 194 men, or 64.88%. Every patient is at least 40 years old. A label of 0 indicates life, and a label of 1 indicates a death event. The dataset is complete, with all records included. The HF phases are categorized as III and IV by the New York Heart Association (NYHA). Every patient had a history of HF and left ventricular systolic dysfunction. The dataset description of the heart failure dataset is given in Table 4.

**Table 4:** Details of attributes in the heart failure dataset.

Features	Measurement	Range
Age	Years	40–95
Anemia	Boolean	0.1
High blood pressure	Boolean	0, 1
Creatinine phosphokinase	Mcg/L	23–7,861
Diabetes	Boolean	0,1
Ejection fraction	Percnetage%	14–80
Sex	Binary	0,1
Platelets	kiloplatelets/mL	25.01–850
Serum creatinine	Mg/dL	0.50–9.40
Serum sodium	mEq/L	114–148
Smoking	Boolean	0,1
Time	Days	4–285
Target Death Event	Boolean	0,1

### Data pre-processing and visualization

The following essential procedures are part of our extensive data pre-processing pipeline, which we use to guarantee dependable and high-quality input for the suggested heart disease prediction model:

#### Outlier removal using improved K-means clustering (IKC)

Outliers have the potential to skew the model’s learning process, particularly when dealing with sensitive medical data. Based on cluster behavior, we identify and eliminate outliers using the Improved K-Means Clustering (IKC) technique^40^. Iteratively, the dataset is divided into clusters, and the cluster centroid is determined. The compactness and separation of clusters are assessed using the Silhouette Index. Outliers are clusters with the smallest population or considerable deviation. To reduce noise from the dataset brought on by inaccurate sensor readings or labels, these outlier clusters are eliminated. By doing this, over-fitting to extreme or unusual data points is avoided, and model robustness is increased.

#### Data normalization using min-max normalization

We employ Min-Max Normalization, which rescales the data to a predetermined range [0, 1], to put all features onto a comparable scale.

1 $$\:{X}_{norm}=\frac{x-{x}_{min}}{{xmin}_{max}}$$

Where is the value in the original dataset. The maximum and minimum values of *x* are denoted as $\:{x}_{max}$ and $\:{x}_{min}$.

In addition to simplifying and speeding up the model’s learning process, this normalization procedure guarantees that no feature is overpowered by its scale.

### Class imbalance handling using SMOTE

**Fig. 2:**
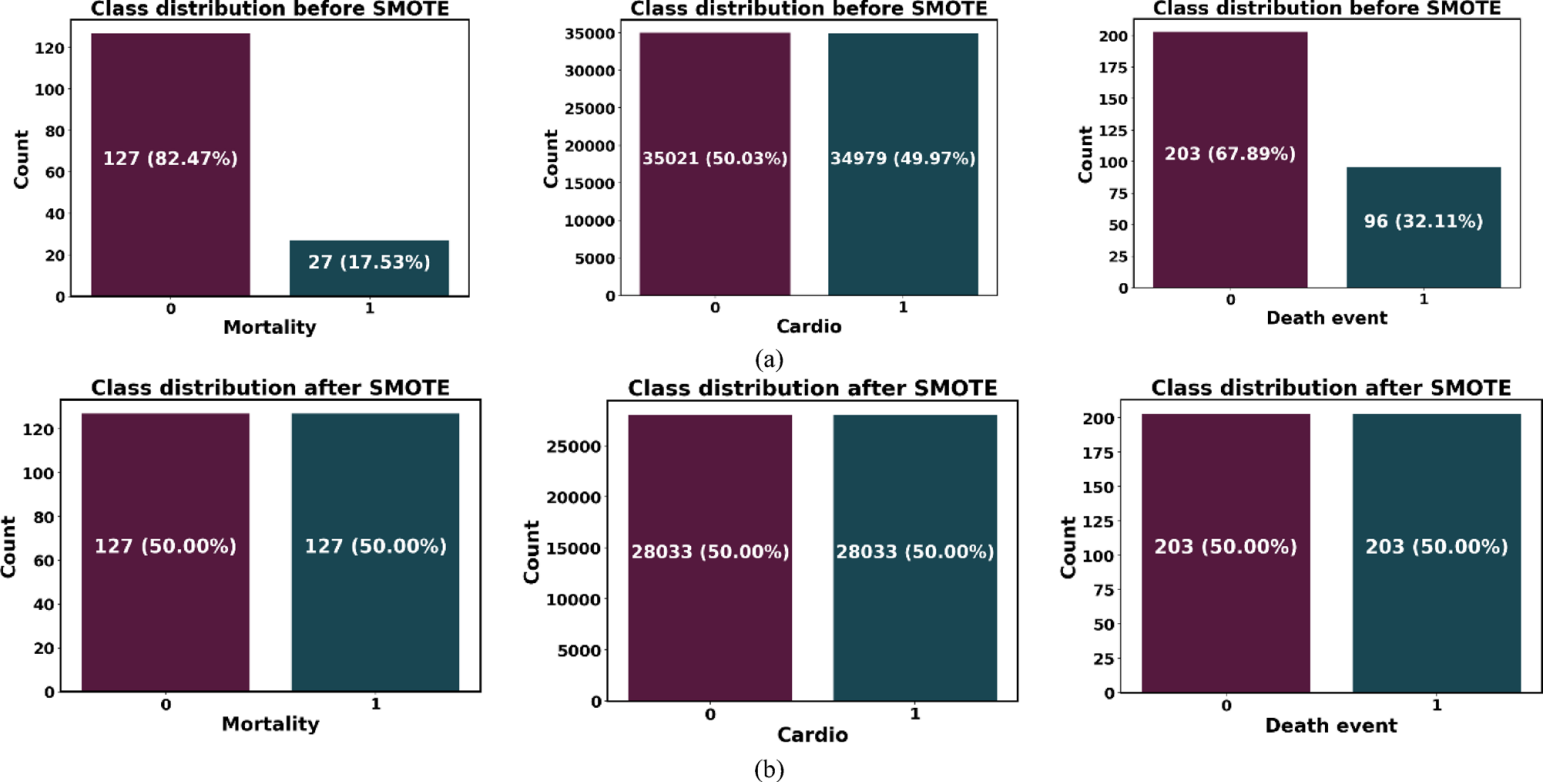
(**a**), (**b**) Visual results of the class distribution in the used dataset, with pre-processing (i.e., outlier removal and balancing) applied to Faisalabad, CVD, and heart failure from left to right.

In medical datasets, where there are usually significantly more healthy cases than ill instances, class imbalance is a common problem. This disparity may cause the classifier to be biased in favor of the majority class, which would reduce its sensitivity in identifying important minority (disease) cases. The Synthetic Minority Over-sampling Technique (SMOTE), which creates synthetic samples for the minority class instead of replicating preexisting ones, was employed to remedy the issue. SMOTE increases the variety of minority class samples without adding redundancy by interpolating between a minority instance and its closest neighbors in feature space. This method was especially crucial for the Faisalabad dataset in our study because it showed the most imbalance. As seen in Fig. 2b, the use of SMOTE produced a more equitable distribution of classes. This pre-processing phase decreased the possibility of incorrectly identifying individuals with heart disease and greatly enhanced the model’s capacity to learn precise decision limits.

#### Data augmentation

In addition to SMOTE, data augmentation techniques were used to improve model generalization and address the dataset’s limited size. SMOTE does not add feature variability beyond interpolation, even though it successfully balances the distribution of classes by synthesizing minority class instances. As a result, we added the following methods to our augmentation strategy:

SMOTE: used to balance class proportions in all datasets, but particularly in the severely unbalanced Faisalabad dataset (Fig. 2).

Random Noise Injection: Minority class samples’ characteristics were subjected to a small amount of Gaussian noise to increase variability without compromising semantic significance.

Feature Perturbation: To resemble real-world measurement variations, some numerical properties were slightly altered within a predetermined range (e.g., ± 5%).

Bootstrapping: In order to improve training resilience and resample small datasets without over-fitting, bootstrapping was employed.

When working with restricted medical records, these techniques can increase the diversity of training samples, avoid over-fitting, and synthetically enlarge the dataset.

As previously stated, Fig. 2a clearly displays class imbalance, especially in the datasets for heart failure and Faisalabad. Figure 2b shows well-balanced class distributions across all datasets following the application of SMOTE and outlier removal, confirming the efficacy of the augmentation technique used.

#### Data visualization

Understanding the correlations between various features (or variables) in a dataset can be facilitated using a correlation matrix, which can be an effective tool in the proposed heart disease prediction research. We can determine which features are more strongly correlated with the existence or absence of heart disease by examining the correlation matrix. Predictive models may prioritize features that strongly correlate with the target variable since they are more likely to be predictive.

#### Data splitting

We separated the data into segments for testing and training. We designated 80% for training and 20% for testing to use the deep learning network model and produce predicted results on unknown data. From the training set, 10% of data are used for the validation process to validate the performance of the proposed approach. The validation set (10% of the data) is specifically used for hyperparameter tuning and model selection. This allows the model to be optimized during training without influencing the test set performance. Because the test set is kept separate from the training and validation sets, there is less chance of data leakage, which could result in performance estimates for heart disease prediction that are too optimistic. To minimize over-fitting and maximize the model’s capacity to generalize to new, unknown data, the random data splitting strategy used in the proposed research provides an organized method for building, fine-tuning, and assessing models. This makes the technique extremely useful for heart disease prediction tasks. The deep learning-based classifier used in this research is a well-respected technique frequently applied to various learning challenges.

#### Statistical feature extraction

To better depict the underlying data distributions, our proposed methodology used statistical features that were directly obtained from the pre-processed dataset attributes. We calculated standard descriptive statistics, such as kurtosis, skewness, variance, standard deviation, mean, minimum and maximum values, and interquartile range (IQR) for every numerical feature. Python’s pandas and scipy.stats libraries were used to extract these statistical features. This procedure enhanced the feature space’s capacity for discrimination and helped to capture the distributional behavior of each attribute. The EWOA was then used to pass these features on to the feature selection phase.

### Feature selection

Bad generalization results from employing irrelevant features when training classifiers, and complexity only increases when redundant features are used. Consequently, determining the best representative subset of features for the prediction task can decrease over-fitting and model complexity while enhancing prediction accuracy and cutting computation time. In this research, the Enhanced Whale Optimization Algorithm (EWOA) is utilized to choose the significant dataset features. In the Faisalabad dataset, the EWOA selects the significant disease-related features Chest Pain Type (CPT), Serum Cholesterol (SC), Resting Blood Pressure (RBP), Maximum Heart Rate Achieved (MHR), Old Peak (OP), Slope, and EIA. In the CVD dataset, the significant features such as Age, Cholesterol level, Smoking status, Body Mass Index (BMI), Systolic Blood Pressure (SBP), Glucose Level, Physical Activity, Gender, Diabetes status, Alcohol consumption are selected for heart disease prediction. In the heart failure dataset, the significant features such as Ejection Fraction (EF), Serum Creatinine, age, Diabetes, High Blood Pressure (HBP), Creatinine Phosphokinase (CPK), Anaemia, Serum Sodium, and Platelets for enhancing the classification performance in terms of accuracy.

The EWOA is a bio-inspired optimization method that draws inspiration from humpback whale hunting behavior. WOA balances between exploitation (finding new solutions) and exploration (finding and refining old ones), imitating the whales’ bubble-net hunting approach. Because of this, it may effectively avoid local minima and identify the ideal subset of features in high-dimensional data, such as those used to predict heart disease. Many variables are frequently included in feature sets for heart disease prediction (age, cholesterol levels, blood pressure, etc.). Large search spaces can be handled well by WOA’s dynamic and adaptive search method, which also lowers the amount of redundant or ineffective features. Non-linear and intricate interactions between features are frequently seen in datasets used to predict heart disease. Because WOA is versatile, it can handle complex goal functions for choosing the most relevant features and can adjust to such non-linearity. When compared to other evolutionary algorithms such as PSO or GA, WOA is computationally less expensive. With everything considered, the EWOA feature selection algorithm provides a quick and versatile means of choosing features for the prediction of heart disease, striking a balance between exploitation and exploration while keeping computing costs low, enhancing model performance, and integrating with different classifiers with ease.

A meta-heuristic algorithm called the WOA^41^ simulates whales searching for prey, surrounding prey, and preying on a spiral bubble net around prey. The humpback whale’s social structure served as inspiration. From four perspectives, WOA was enhanced: position update, parameter correction, probability selection, and population initialization to increase the convergence speed and accuracy of WOA.

By addressing frequent flaws in conventional feature selection methods that might cause accuracy decreases, the EWOA enhances feature selection for heart disease prediction.

EWOA improves the ratio of exploitation (fine-tuning the search in promising areas) to exploration (scanning the global feature space). This lessens the possibility of becoming trapped in local optima, which can cause issues with other algorithms such as GA and PSO.

Because heart disease datasets may contain duplicate or unnecessary characteristics, EWOA constantly modifies its search behavior based on the current feature population. This enables it to adapt to the complexity of these datasets. This is especially useful for medical data since the relative value of features might change greatly.

Premature convergence is a problem with traditional feature selection methods, where the algorithm converges too early on an inferior feature set. To prevent this, EWOA uses techniques like non-linear control schemes, making sure that crucial aspects are not overlooked.

Due to its faster convergence and greater capacity for feature subset optimization, studies have demonstrated that EWOA outperforms other feature selection techniques such as GA, PSO, and even the traditional Whale Optimization Algorithm (WOA) in terms of classification accuracy.

Datasets used for cardiac disease are frequently unbalanced or noisy. Because of its resilience, EWOA can manage this type of data more effectively, choosing feature sets that preserve good prediction performance without either over- or under-fitting.

### Classification

In this research, the presence and absence of heart disease are classified using an improved hybrid deep-learning approach. The improved InceptionCapsule network model is used for heart disease classification. Cutting-edge results have been shown by the InceptionCapsule method on several classification tasks, such as object detection, image classification, and medical data analysis. The hybrid Inception-Capsule model is a more effective choice than DenseNet, AlexNet, or ResNet in these cases, where accuracy, interpretability, and robustness are paramount.

The hybrid Inception-Capsule model, while powerful, faces two challenges that can affect its performance, especially in complex tasks like heart disease prediction. Computational complexity and routing inefficiency are two of the major problems of the basic InceptionCapsule network. These two issues are resolved by improving the InceptionCapsule method. This method takes initial weights from ImageNet and avoids random weight selection using transfer learning and the Inception-ResNet model. Additionally, it creates rich vectors utilizing the output of the Inception middle layers. Vectors that have been extracted are fed into a learning capsule network that has an attention mechanism.

**Fig. 3:**
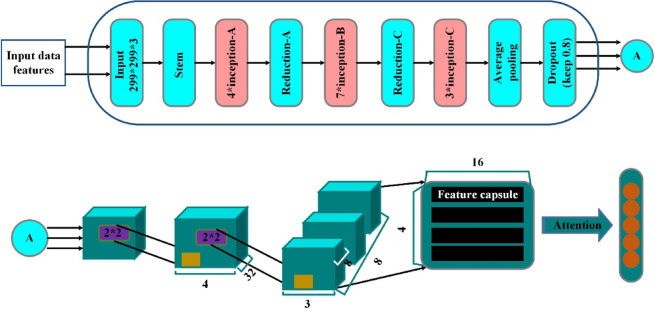
The structure of inception networks.

**Fig. 4:**
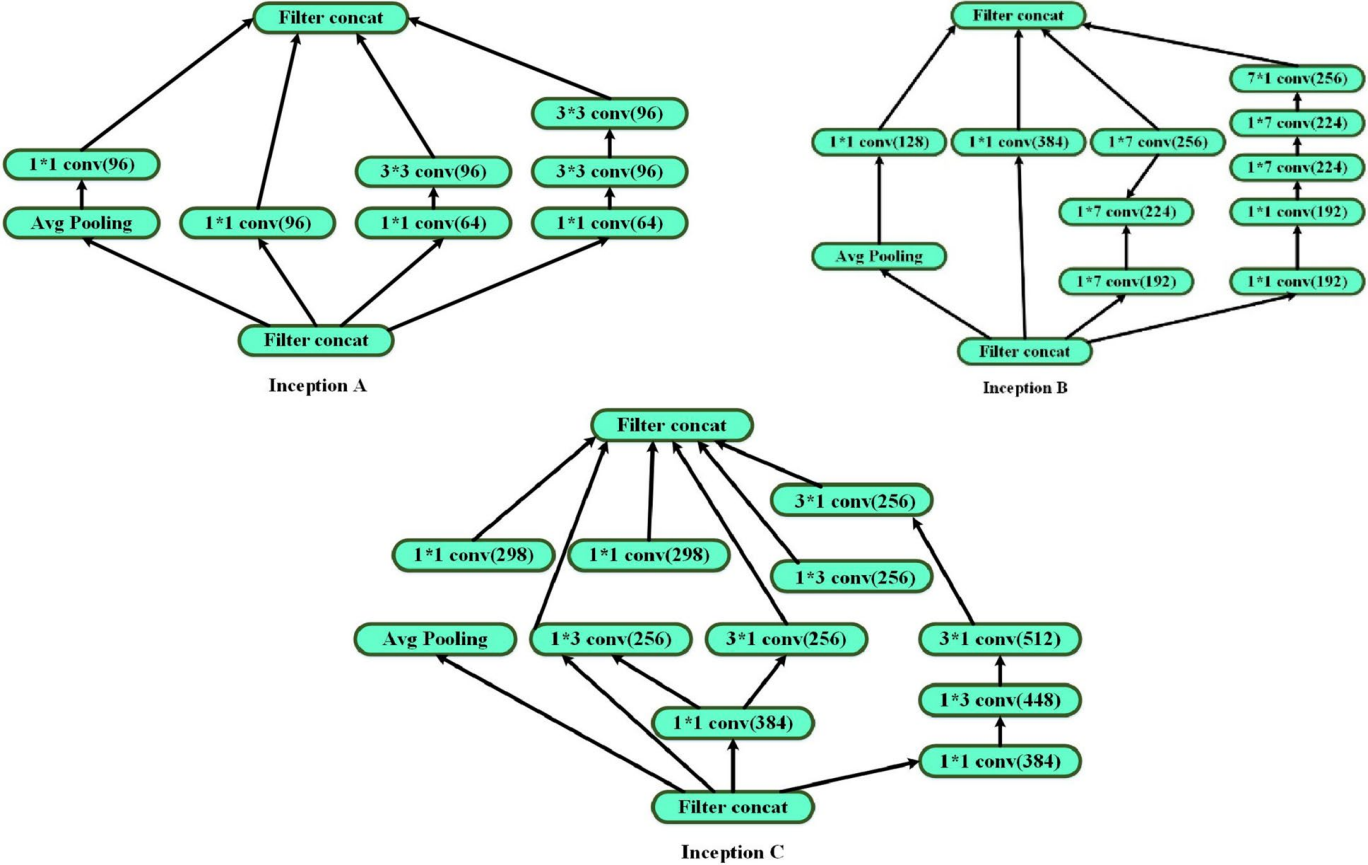
The proposed IDLHICNet architecture.

Using this Inception-ResNet network, we accomplish the feature extraction task in our method. Even with the advances in deep learning techniques, they still need to produce accurate classifications when the model is scaled, which results in over-fitting and poor performance. In other deep learning domains, one effectively implemented model, the Inception-ResNet, is the foundation for our proposed InceptionCapsule model. It aims to achieve a more accurate classification task, with the Inception module and the residual connections as its main components. From the Inception-ResNet model, this Inception capsule model is created. The TensorFlow Keras API is used to implement the Inception module, as shown in Fig. 3. The Inception structure including Inception-A, Inception-B, and Inception-C is shown in Fig. 4.

## Result and discussion

For determining the probability of survival for patients with heart disease, the exploratory methodology and research findings are examined in this section. Using the significant features to determine if a patient has heart disease or not for the binary classification task, the results with all attributes are shown. The dataset is balanced using the SMOTE tool, and the deep learning-based classifier’s prediction scores are enhanced through hyperparameter optimization. The hybrid deep learning model is then trained using the balanced dataset, and assessments are made for F1-Score, recall, precision, and accuracy.

### Experimental settings

Using the IDLHICNet model, the performance of the algorithms was examined. The deep learning model is made using the Scikit-Learn library module and the Python programming language. An 80:20 ratio is used to split the data between the training and testing stages. To evaluate the significance of the proposed approach, a range of performance evaluation metrics are utilized. Using the testing performance evaluation, the efficacy of the proposed hybrid heart disease prediction model is examined across all datasets. Each experiment was carried out on a system running Python 3.10 with TensorFlow 2.12, an Intel Core i7 CPU operating at 3.20 GHz, 32 GB of RAM, and an NVIDIA RTX 3060 GPU.

### Hyper-parameter settings

Using the Adam optimizer to optimize a CNN model’s hyper-parameters entails methodically adjusting several important parameters to enhance model performance. Hyper-parameters influence variables such as convergence speed, model accuracy, and generalization, and they regulate the learning process. A gradient descent variation called Adam (Adaptive Moment Estimation) dynamically modifies the learning rate in response to previous gradients. It makes use of two parameters: β2 (for the second-moment estimate or RMSProp-like behaviour) and β1 (for the first-moment estimate or momentum). The Adam optimizer is used to fine-tune the hyper-parameters of the proposed network model. The Adam optimizer typically tunes the Batch size, epochs, dropout rate, momentum, weight decay, and momentum. Since batch size determines how many training samples are used in an iteration before updating the model’s weights, we decided on 32 based on the proposed model’s memory capacity. We select a total of 100 epochs since this is the number of times the complete training set is processed through the network. Selecting the appropriate number of epochs is important to prevent either over-fitting or under-fitting. During training, neurons are randomly “dropped out” as part of the dropout approach, which helps prevent over-fitting., a dropout rate of 0.5 is chosen. With a learning rate of 0.001, Adam automatically modifies the rate of learning for every parameter based on estimates of the first and second moments of the gradients. This allows the model to converge more slowly, which is advantageous for stability and fine-tuning. We use a decay of 0.01 because it is more appropriate for brief training schedules. Learning rate decay lowers the learning rate over time, allowing the model to converge more slowly as it approaches the better solution. The optimized parameters are given in Table 5.

**Table 5:** Optimized parameters of the classification network.

Hyper-parameters	Values
Activation function for the output layer	Softmax
Batch size	32
Training epochs	100
Dropout rate	0.5
decay	0.01
Momentum	0.9
Learning rate	0.001

### Performance measures

For heart disease classification, the five metrics are analyzed using the proposed approach based on the experimental results: F1-score, recall, precision, accuracy, and AUC. The following equations explain the performance metrics.

Accuracy: Comparing the total predicted values with the number of predictors needed for a successful classification is an accuracy test.

2 $$\:Accuracy=\frac{TP+TN}{TP+TN+FP+FN}\:$$

Precision: It calculates the ratio of the total predicted values to the positive attributes.

3 $$\:Precision=\frac{TP}{TP+FP}$$

Recall: It is employed in the computation of the average mean accuracy to recall ratio.

4 $$\:Recall=\frac{TP}{TP+FN}$$

F1-Score: The mean average accuracy to recall ratio is calculated using the F1-score.

5 $$\:F1-Score=2*\frac{precision*recall}{precision+recall}$$

AUC: The Area under the ROC Curve (AUC) is used to evaluate how well the model differentiates between positive and negative classes. A greater AUC value, calculated using Eq. (6), denotes overall model performance and is indicative of a high sensitivity (i.e., rate of correctly detected positive cases) and a low rate of false positives.

6 $$\:AUC=\frac{1+TPR-FPR}{2}$$

### Experimental results of the Faisalabad dataset

**Fig. 5:**
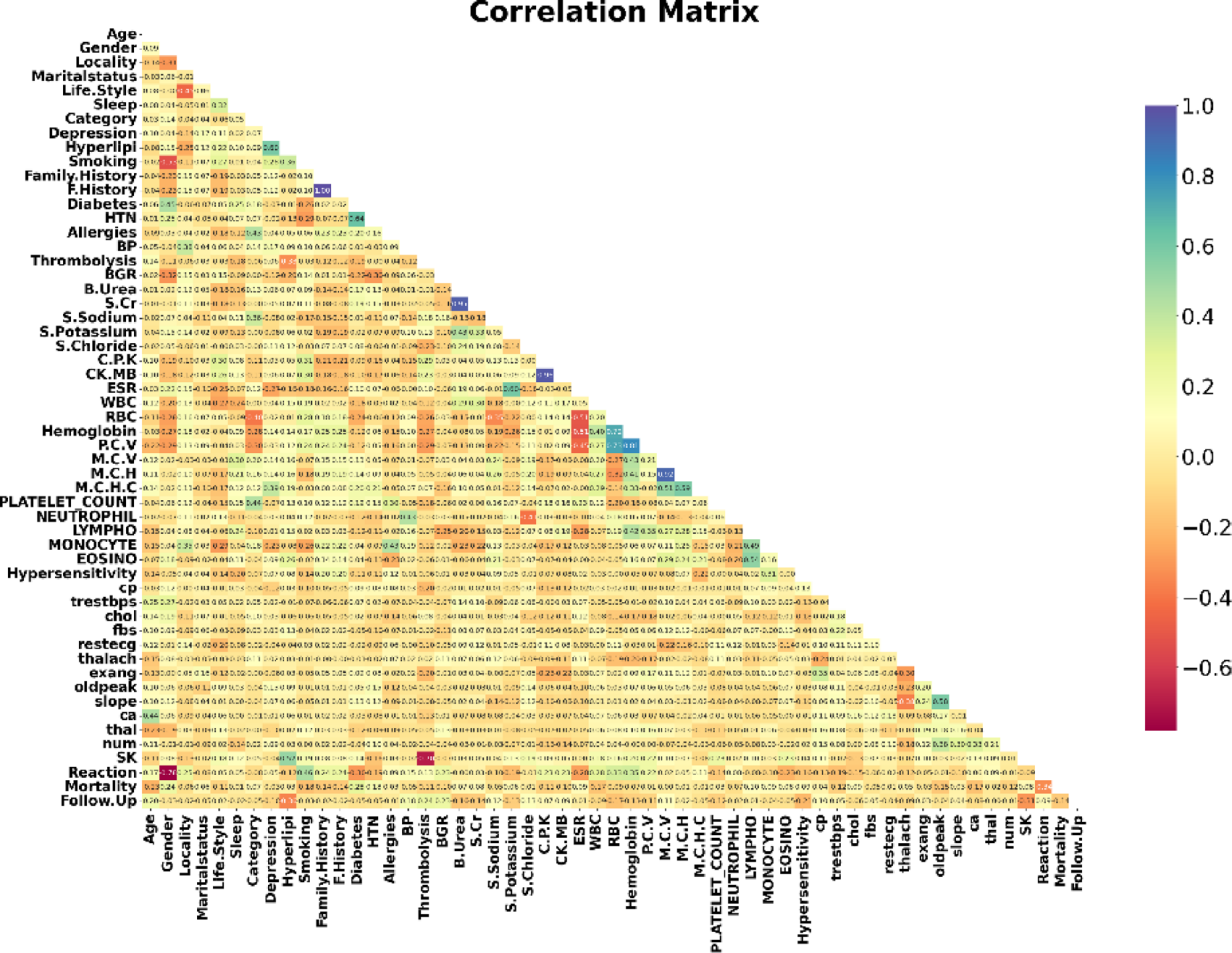
Heatmap distribution of the dataset features for the Faisalabad dataset.

For the Faisalabad dataset, a correlation heatmap is first made, as seen in Fig. 5.

**Fig. 6:**
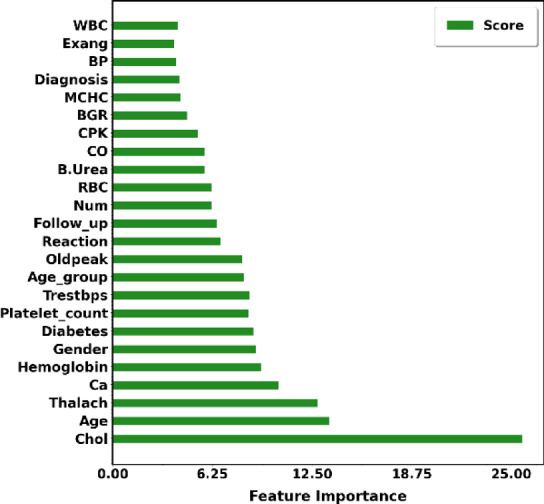
Ranking of attributes selected by EWOA feature selection algorithm for CVD prediction on Faisalabad dataset.

Using the Faisalabad dataset, the feature selection method produced the primary attributes related to heart disease. These features are listed in ranking order in Fig. 6. Features including age, cholesterol, and thalach are strongly correlated with the probability of developing heart disease.

**Fig. 7:**
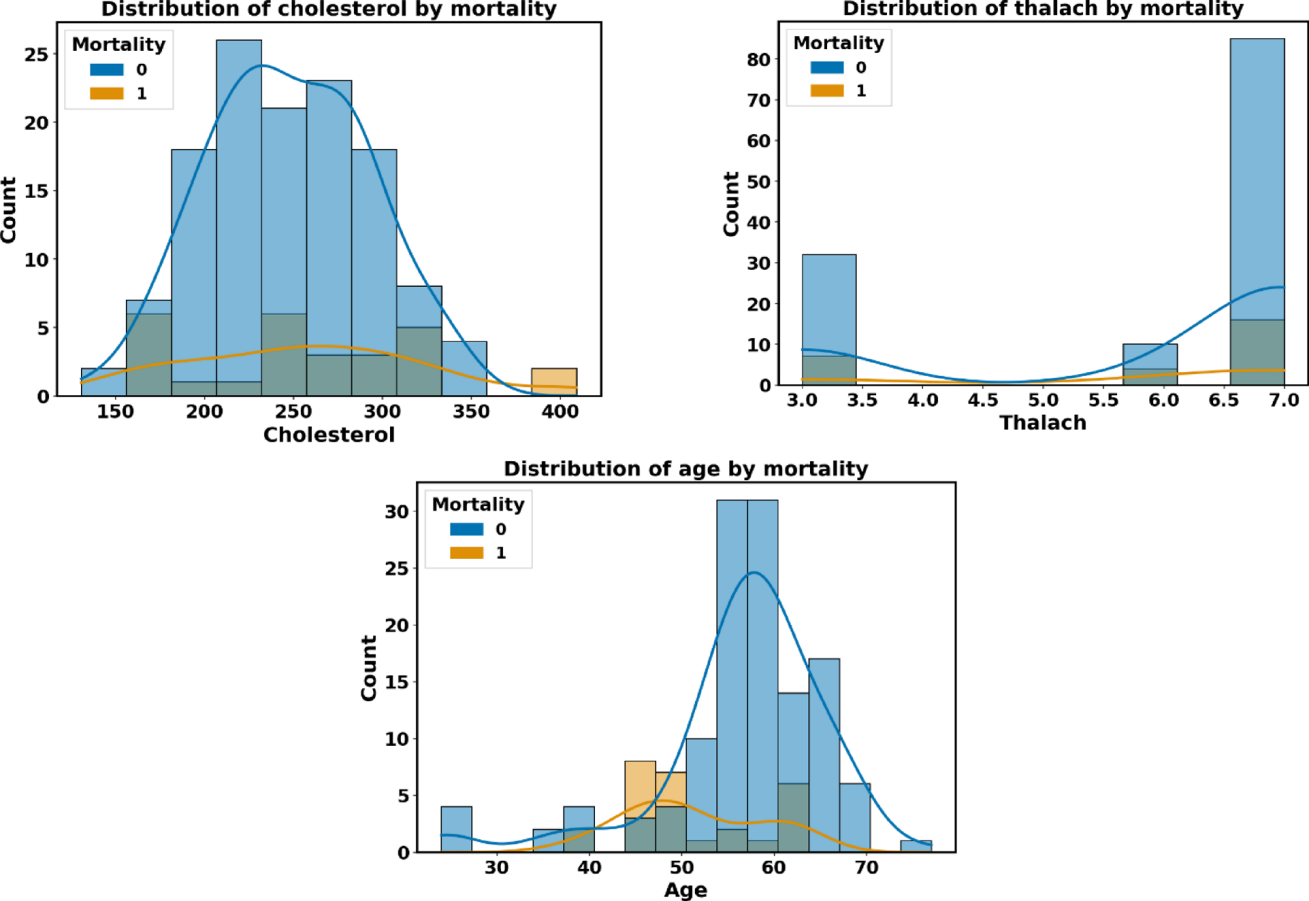
The Faisalabad data distributions of input features by target feature T.

The input features of the Faisalabad dataset are visually shown in Fig. 7 about the target feature T. The target feature distribution of each significant feature is represented by the horizontal axis, while the vertical axis shows the value count. The frequency distribution of the values for each feature is represented by the histogram in each plot, while a smooth estimation of the data distribution is shown by the kernel density estimation.

**Fig. 8:**
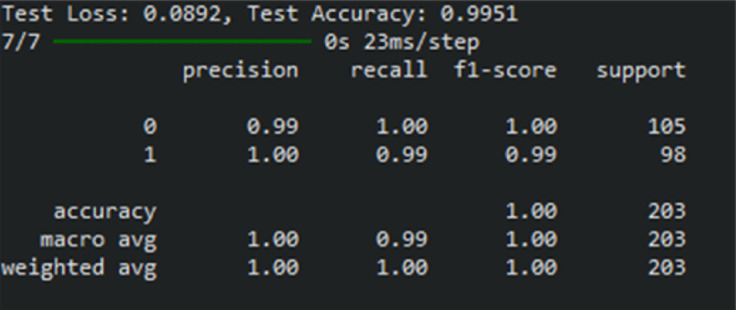
The classification report of the developed heart disease prediction model for a Faisalabad dataset.

Figure 8 provides the classification report of the proposed heart disease prediction for the Faisalabad dataset in both normal and abnormal cases. The proposed model classifies 98 patients as abnormal and 104 patients as normal. According to these results, the proposed approach can diagnose heart disease with a 99.50% F1 score, 99.50% recall, 99.53% precision, and 99.51% classification accuracy. For predicting heart disease, the proposed approach is the most effective, as indicated by these measures. The proposed results in fewer false positives and false negatives, indicating good classification accuracy.

**Fig. 9:**
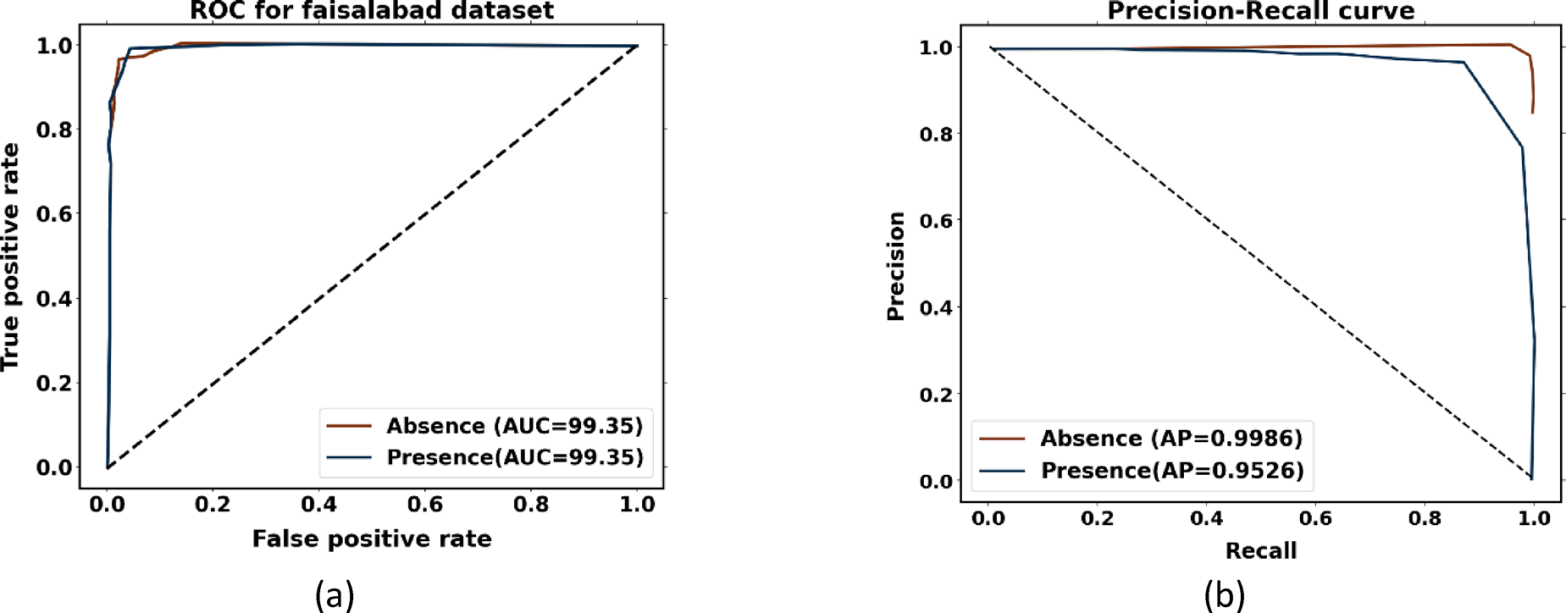
Performance analysis curves for heart disease prediction using Faisalabad dataset (**a**) ROC curve and (**b**) precision-recall curve.

To examine the overall effectiveness of the proposed approach for the Faisalabad dataset, Fig. 9a presents the ROC curve visualization. With AUC scores of 0.9935 for the absence of heart disease and 0.9935 for the presence of heart disease, the proposed approach performs better than the other models. AUC values greater than 99% for both normal and abnormal classes indicate the presence of an optimal model. Figure 9b displays a precision-recall curve with recall plotted on the x-axis and accuracy on the y-axis. An average precision (AP) of 0.9986 indicates absence, and 0.9526 indicates presence; it has been found.

### Experimental results of Kaggle’s CVD dataset

**Fig. 10:**
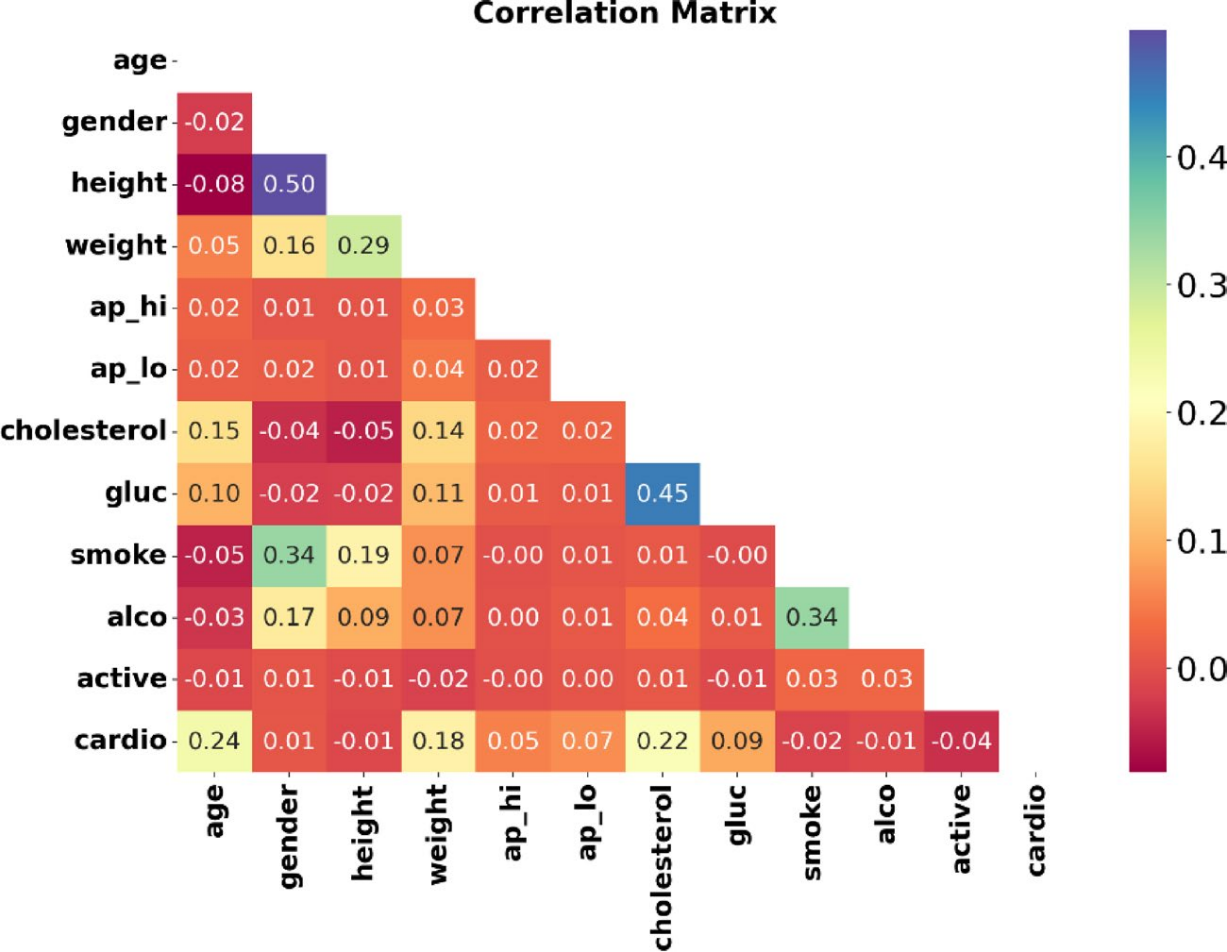
Correlation values of all features.

In Fig. 10, the features of the CVD dataset are evaluated using a correlation heatmap. From Fig. 10, gender, PP, glucose, MAP, BMI, and smoke are highly correlated factors.

**Fig. 11:**
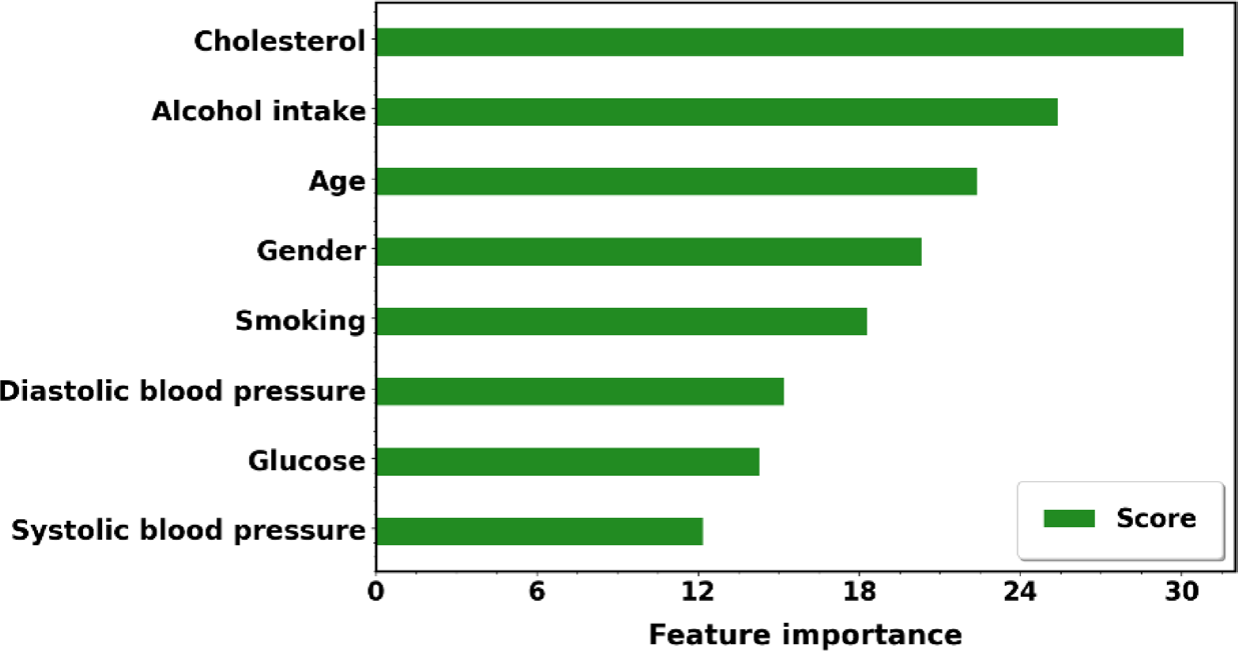
Feature importance according to the EWOA feature selection algorithm for the CVD dataset.

According to a classifier’s perspective, a feature’s significance for forecasting the target variable is indicated in Fig. 11. Features of alcohol and cholesterol intake are highly associated with the prognosis of heart disease.

**Fig. 12:**
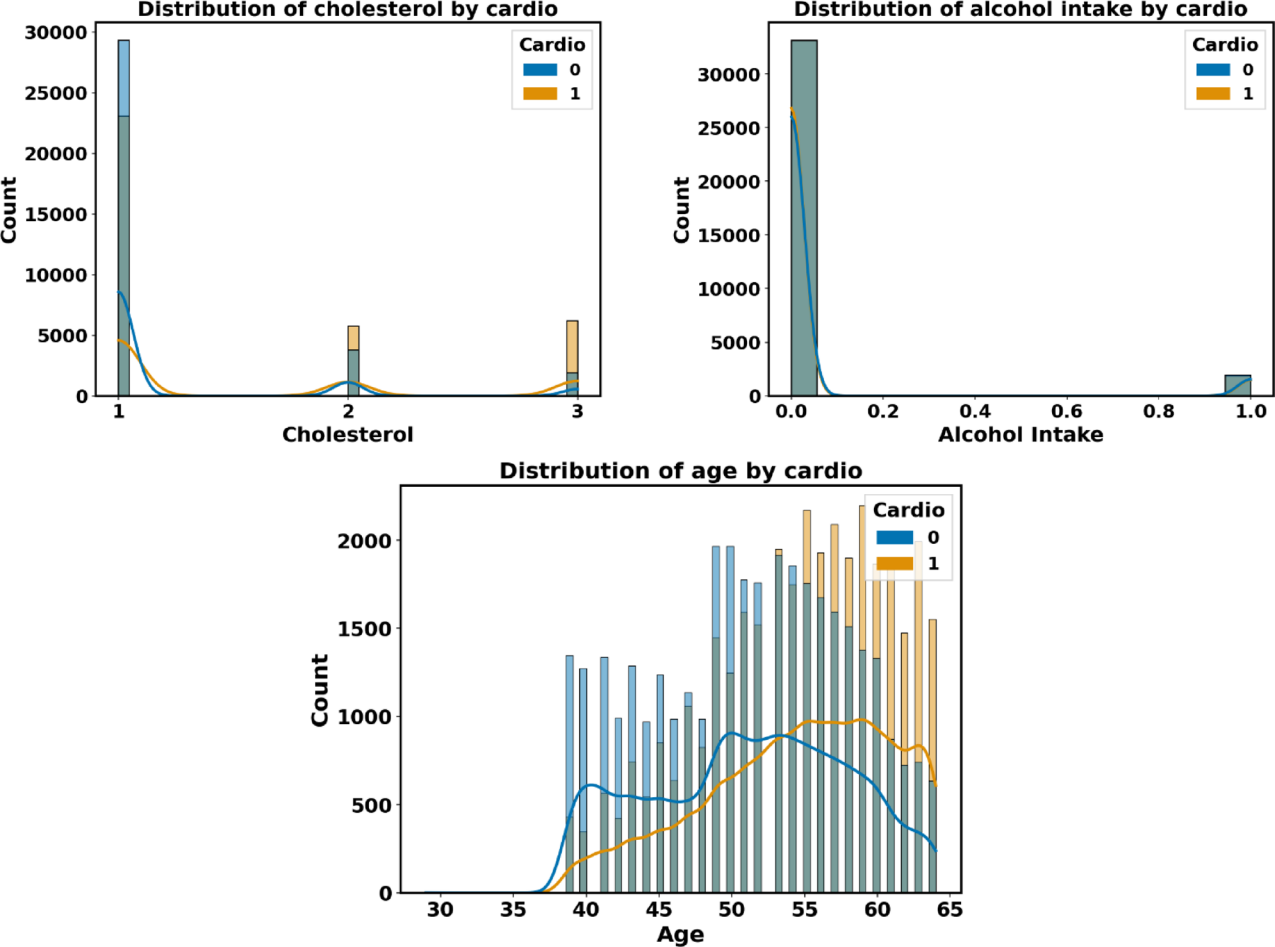
The CVD data distributions of input features by target feature T.

Each distribution feature for the CVD dataset is represented in Figure 12 with persons with and without heart disease. Heart disease patients are accurately predicted using the proposed heart disease prediction approach. Healthcare practitioners can identify people who are at risk of getting heart disease before symptoms arise by analyzing patient attribute data.

**Fig. 13:**
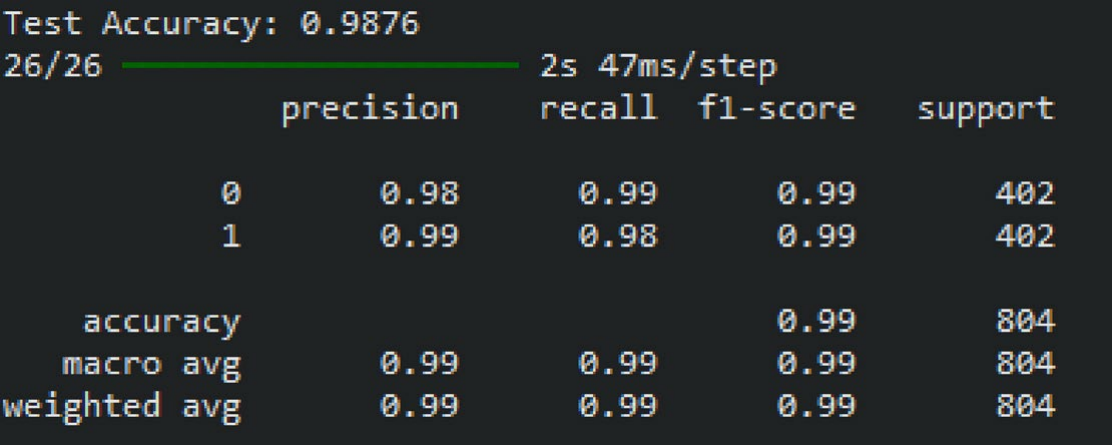
The classification report of the proposed heart disease prediction model for a CVD dataset.

Figure 13 displays the classification report of the proposed heart disease prediction model for the CVD dataset. With a small number of false positives and negatives derived from the confusion matrix, the proposed heart disease prediction model yields better classification results, with an F1-score of 99%, recall of 98.50%, precision of 98.50%, and accuracy of 98.76%. The proposed model accurately classifies 397 patients as normal and mis-classifies 5 as abnormal based on the confusion matrix, whereas it correctly classifies 395 patients as abnormal and mis-classifies 6 as normal.

**Fig. 14:**
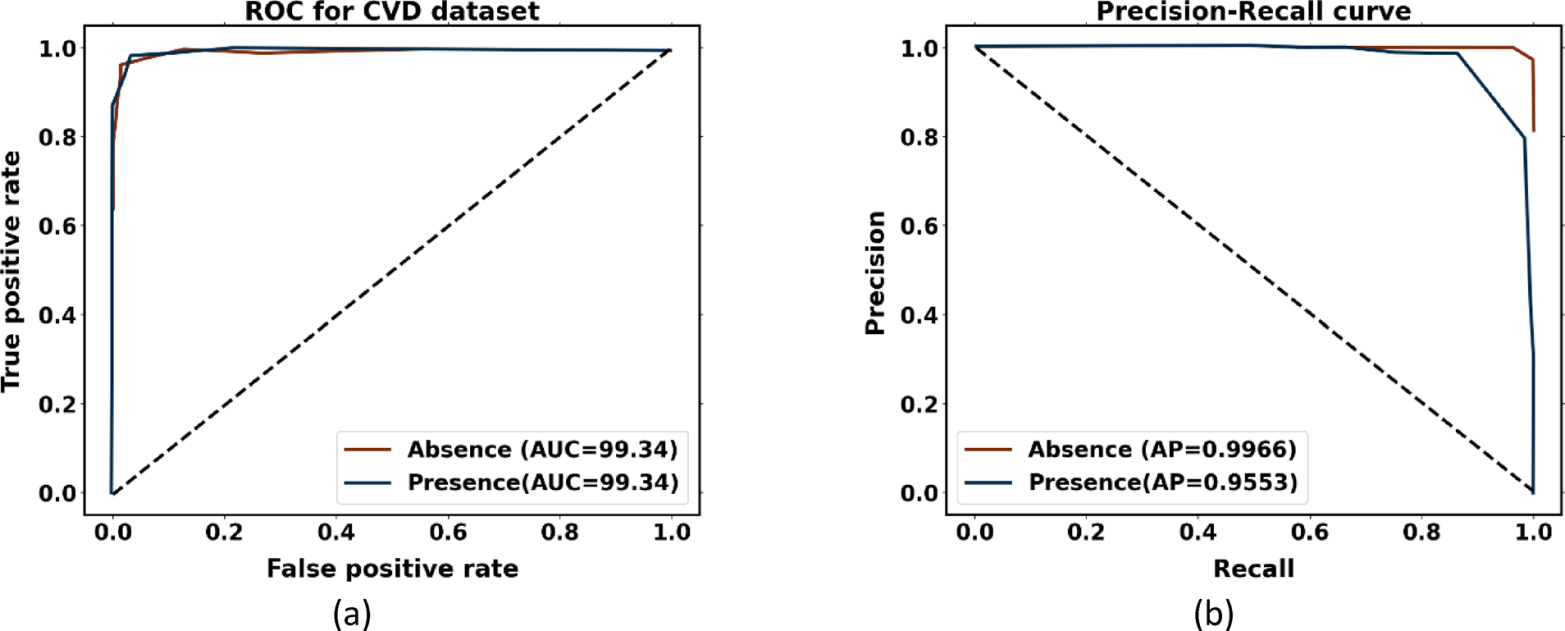
Performance analysis curves CVD dataset (**a**) ROC curve and (**b**) Precision-Recall curve.

The performance analysis curves for the CVD dataset are given in Fig. 14. Figure 14a displays the ROC curve for verifying CVD tests; the AUCs for presence and absence are 0.9934 and 0.9934, respectively. Higher AUC values for normal cases indicate that the model is more adept at differentiating between normal and abnormal occurrences when using the proposed approach. For CVD validation, the precision-recall curve is displayed in Figure 14b. It is discovered that the average accuracy (AP) for presence is 0.9553, and for absence, it is 0.9966. A greater Precision-Recall curve shows that the model minimizes false positives while accurately detecting positive cases or individuals with heart disease.

### Experimental results of heart failure dataset

**Fig. 15:**
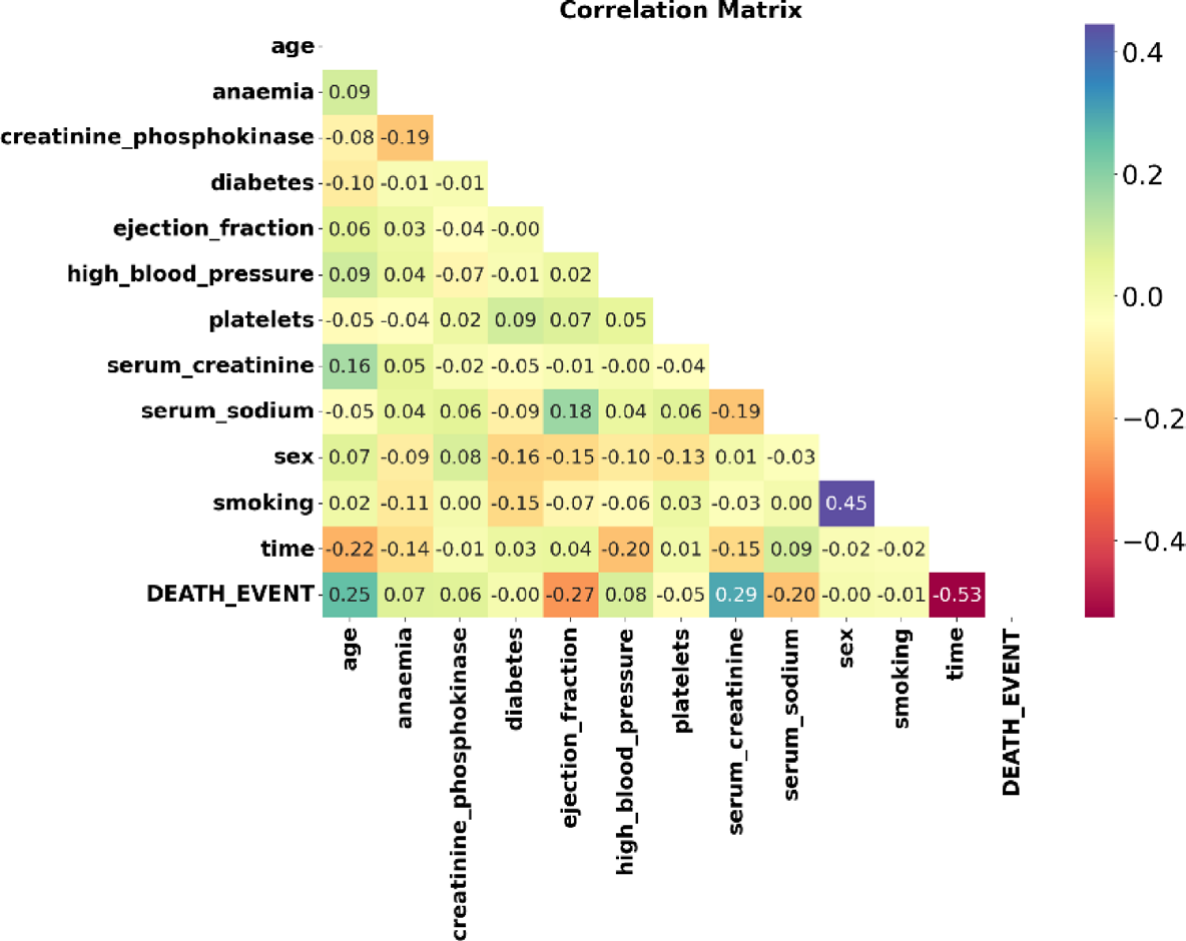
Correlation heat map of heart failure dataset.

All of the data’s input attributes are numerical. The correlation analysis of the attributes in the heart failure dataset is shown in Fig. 15.

**Fig. 16:**
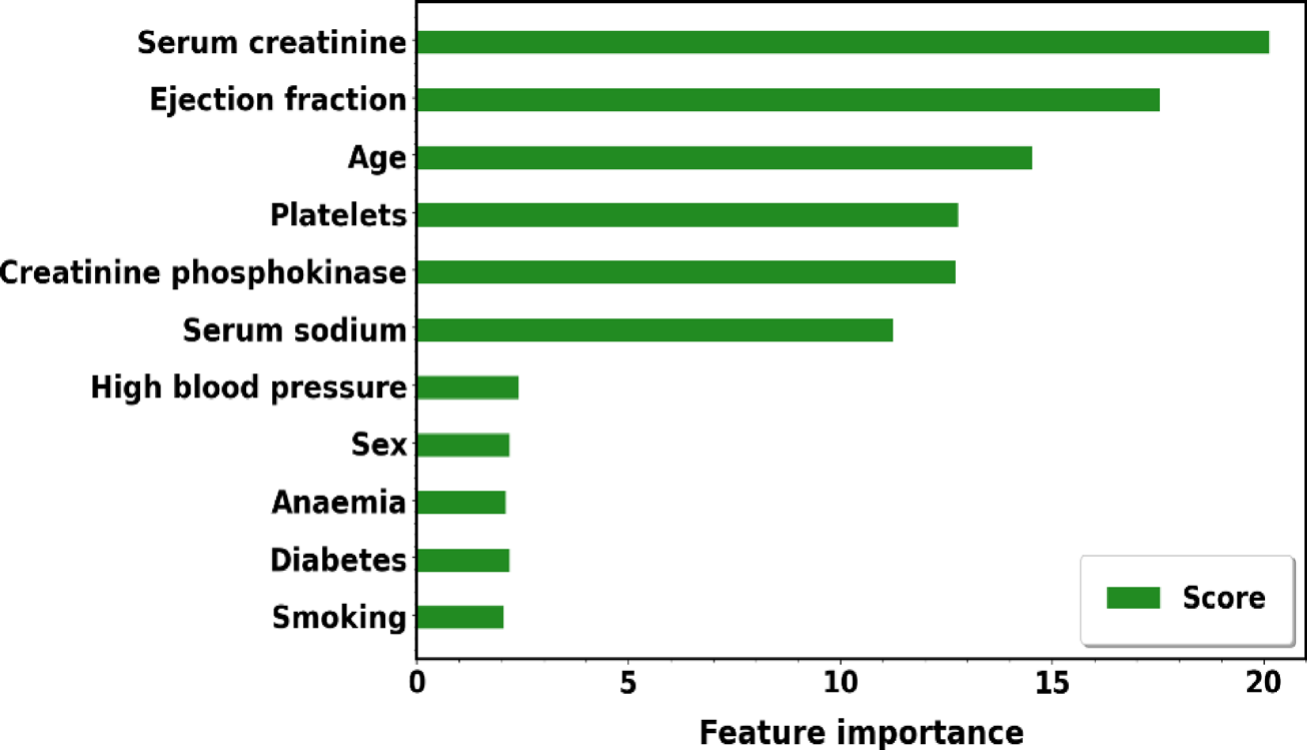
Features from the dataset were chosen using EWOA.

For heart disease prediction, Fig. 16 provides a brief description of the features that EWOA determined to be most important. Features in the bar plot with lower rankings are considered more relevant, considering their greater impact on the prognosis of heart disease. In this Figure, the chosen features lists the features that are retained in the final subset, which may aid in the interpretation of the model as well as guide future research. Using this information, we can focus on a collection of features that are most important for the model’s ability to make predictions, which could improve the model’s ability, interpretability, and efficiency in generalizing new data. Features such as ejection fraction utilization and serum creatinine have a strong correlation.

**Fig. 17:**
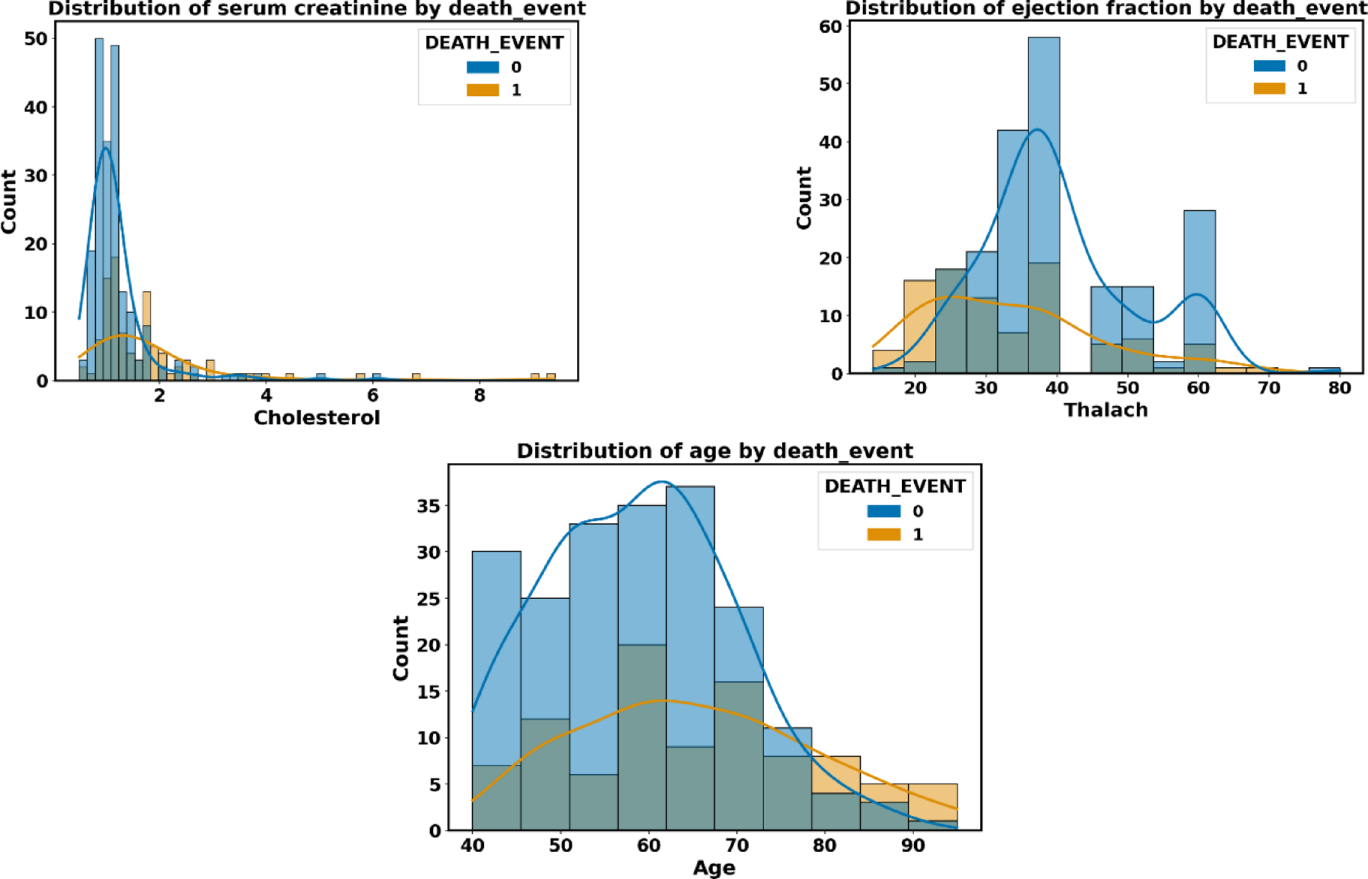
The heart failure data distributions of input features.

The heart failure dataset’s visual representation of input attributes about each distribution is displayed in Fig. 17.

**Fig. 18:**
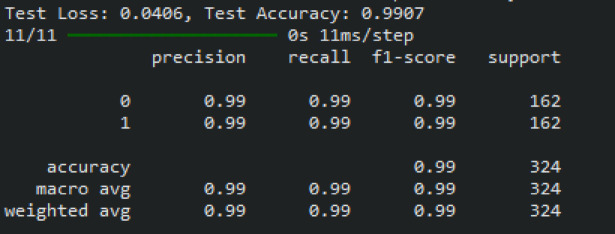
The classification report of the proposed approach for heart disease prediction using the heart failure dataset.

Figure 18 shows the classification report of the proposed approach for the Heart failure dataset. The proposed approach achieves 99.22% F1-score, 99.05% recall, 99.14% precision, and 99.07% classification accuracy. The proposed method accurately classifies 107 persons as normal and 67 as abnormal.

**Fig. 19:**
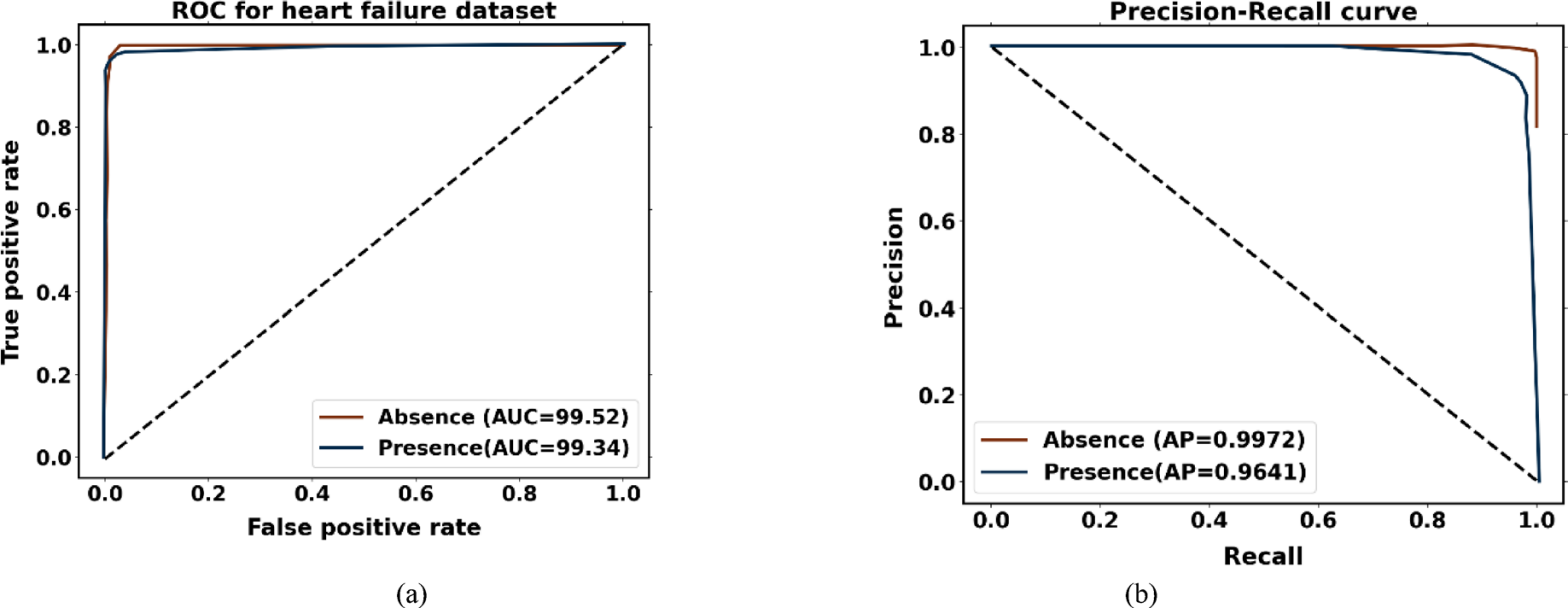
Performance analysis curve for heart failure dataset (a) ROC curve and (b) Precision-Recall curve.

The performance analysis curves for the heart failure dataset are shown in Fig. 19. With the maximal classification accuracy, the proposed approach correctly identifies normal and abnormal classes, as shown by Fig. 19a, where the presence and absence have an AUC of 0.9934 and 0.9952. Based on the precision-recall curve (Fig. 19b), the average precision (AP) for an absence is 0.9972, and the presence is 0.9641. The proposed approach shows a lower rate of inaccurate diagnoses and produces higher prediction outcomes with greater precision. An early diagnosis of heart disease in patients who are at risk is made possible by increased prediction precision. Better disease management and better patient outcomes can result from early intervention.

**Fig. 20:**
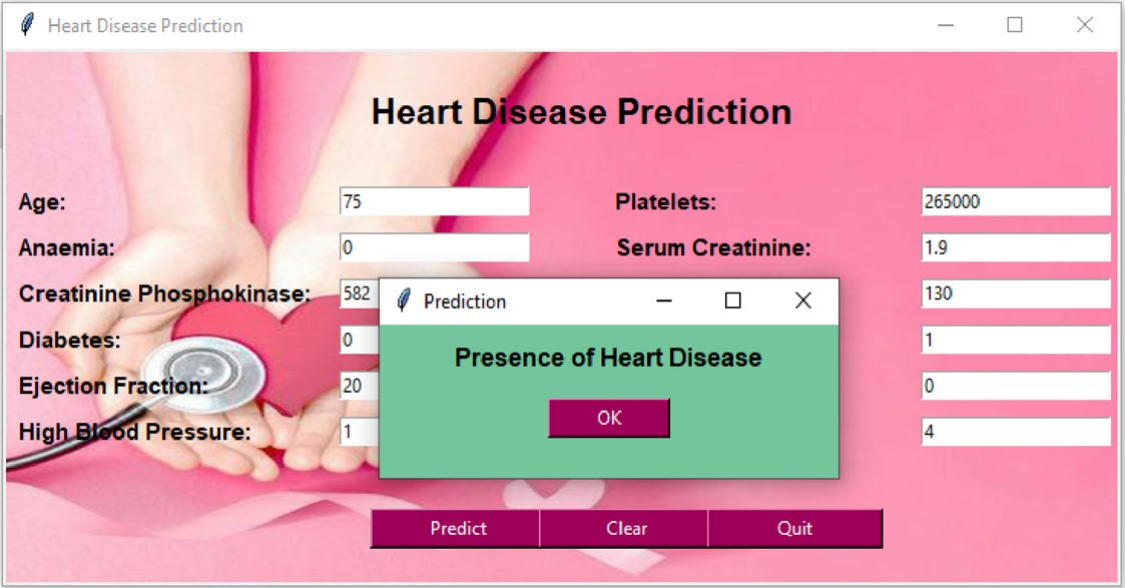
Example GUI for presence of heart disease using heart failure dataset.

Figure 20 shows the two possibilities of the heart disease prediction system, heart disease presence on a graphical user interface (GUI) made using Tkinter in Python.

### Performance analysis of training and validation

**Fig. 21:**
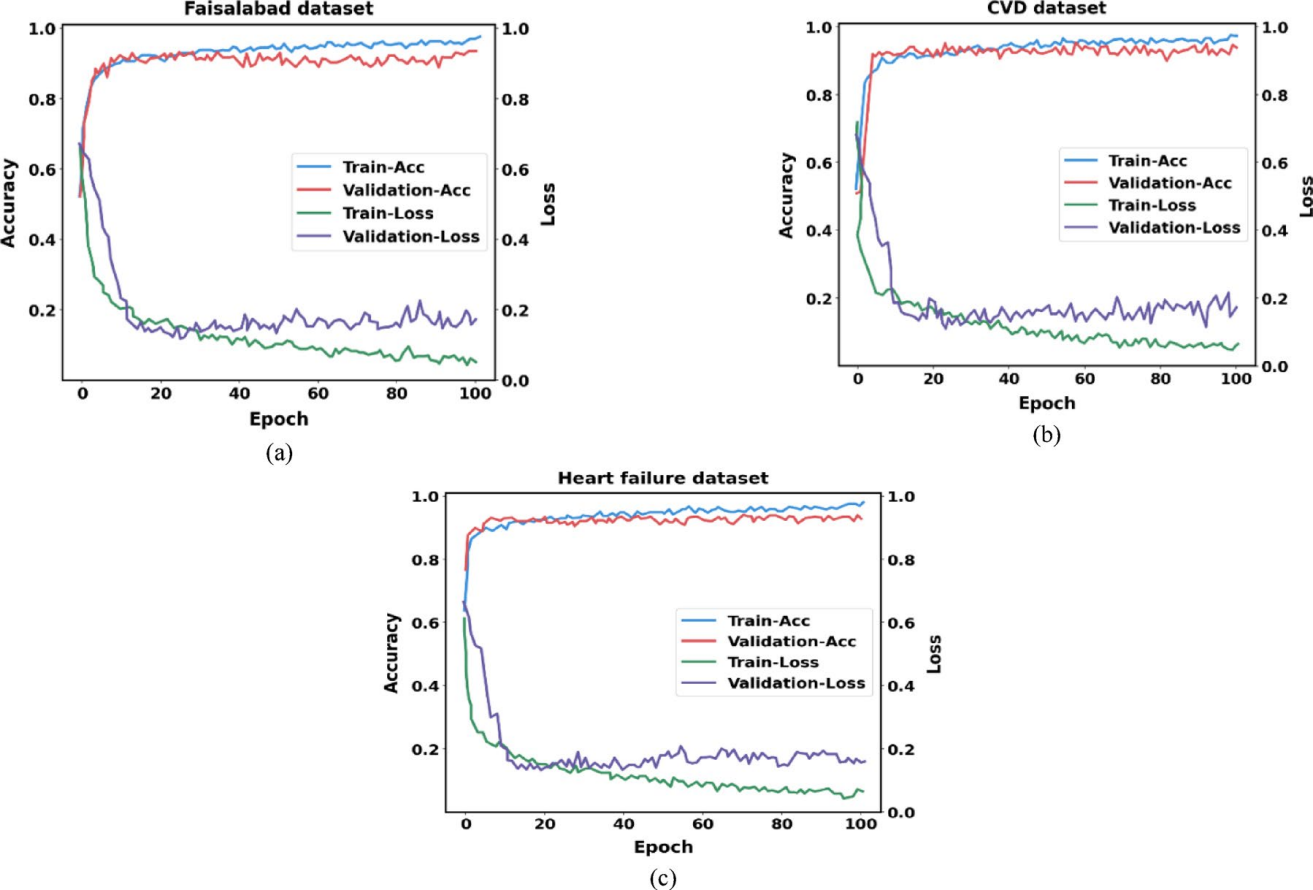
Evaluation of training and validation (Accuracy and loss) (**a**) Faisalabad dataset, (**b**) CVD dataset, and (**c**) Heart failure dataset.

On both the training and validation datasets, our proposed (IDLHICNet) model achieves high accuracy and low loss, demonstrating that it is learning effectively and generalizing well to new data. It is possible to direct the model training process and improve overall performance by identifying issues such as under- or over-fitting and focusing on the correlation between testing and training metrics. The accuracy and loss curves for the Faisalabad, CVD, and heart failure datasets are displayed in Fig. 21 for training and validation. For all datasets, our proposed model’s training accuracy is marginally better than our validation accuracy, and our training loss is marginally less than our validation loss. This is because the model is specifically optimized on the training data, which leads to better performance on that data compared to the test data. There is little difference between the accuracy and loss scores of the training and validation sets. Indicating good generalization effects of the proposed model. Accuracy in training and validation eventually approached 0.99. When comparing all dataset’s training and testing data, the training data displayed higher accuracy. This guarantees that the underlying patterns and relationships in the data are accurately captured by the model. From the accuracy and loss curves, the proposed model is capturing and learning these important features effectively without over-relying on the specifics of the training data. A training loss of 0.1 and validation loss of 0.2 for the Faisalabad dataset, a train loss of 0.1 and validation loss of 0.25 for the CVD dataset, and a train loss of 0.15 and validation loss of 0.2 for the heart failure dataset is achieved by the proposed model. Given that the two curves closely resemble each other and the validation loss is slightly greater than the training loss, it appears that the model and the data are reasonably well-fitted. Our proposed approach appears to have discriminated between the normal and abnormal classes despite having few parameters.

### Performance comparison

The metrics used in the previous research are compared with values from the metrics used in this research in this section’s comparative analysis with other similar papers. The most recent research in the field using the same datasets is the work used. Table 6 lists recent existing papers using the same three datasets utilized in this research. We contrasted the stated metrics of the classification models for heart disease prediction. Thorough testing and comparison with existing models and methodologies show that the proposed IDLHICNet model performs better than others. With the Faisalabad dataset, the CVD dataset, and the heart failure dataset, our IDLHICNet model achieves a greater accuracy of 99.51%, 98.76%, and 99.07%.

**Table 6:** The comprehensive comparison of proposed and existing approaches.

Dataset	Reference	Year	Model used	Accuracy (%)	Precision (%)	Recall (%)	F1-score (%)
Faisalabad dataset	Altantawy et al.^24^	2024	Deep attentive model	98	98.5	98.5	98
	Ogunpola et al.^42^	2024	XGBoost	98.5	99.14	98.29	98.71
	Sutradhar et al.^43^	2023	IBS classifier	92.75	91	94.3	92.61
	Jafar et al.^25^	2023	Gradient Boosting (GB)	91.66	85.02	72.22	83.87
	Proposed model		IDLHICNet	99.51	99.53	99.50	99.50
CVD dataset	Omkari et al.^26^	2024	TLV model	88.9	89.5	89.7	89.6
	Rao et al.^44^	2024	HMSI-AttGRU	95.42	92.51	98.86	95.58
	Bhatt et al.^45^	2023	MLP	87.28	88.7	84.85	86.71
	Wankhede et al.^46^	2022	TSA-EDL	98.33	97.24	98.03	98.41
	Reshan et al.^47^	2023	CNN-LSTM	98.52	98.13	98.11	98.06
	Proposed model		IDLHICNet	98.76	98.50	98.50	99
Heart Failure dataset	Qadri et al.^48^	2024	RF	97.75	98	98	98
	Nandy et al.^49^	2023	Swarm-ANN	95.78	95.21	95.21	-
	Abdellatif et al.^50^	2022	Weighted RF	97.2	94.4	94.3	98.2
	Proposed model		IDLHICNet	99.07	99.14	99.05	99.22

Using the Faisalabad dataset, Ogunpola et al.^42^ employ an XGBoost classifier for the prediction of heart disease; this classifier does not automatically learn complicated or hierarchical features from unprocessed data. This could be a drawback if the dataset performs poorly in heart disease prediction due to complicated patterns that are difficult to capture. However, the complicated patterns connected to heart disease are automatically extracted by our proposed (IDLHICNet) model. As more features are added, XGBoost models may grow more intricate and challenging to understand. On the other hand, our proposed IDLHICNet model employs end-to-end learning to handle complexity more cohesively, despite its complexity.

Using the Faisalabad dataset, Sutradhar et al.^43^ created an Imperial boost-stacked (IBS) classifier for the prediction of heart disease. Because the IBS classifier uses ensemble approaches, the stacking of numerous models can make it extremely complex. The proposed IDLHICNet model can enable end-to-end feature learning and direct predictions from data, potentially simplifying the model structure. However, this complexity can make the model challenging to interpret. IBS classifiers tend to overfit since they may collect noise in the data instead of patterns that can be applied to a larger dataset. By utilizing regularization approaches, capsule networks, when combined with Inception networks, can lessen over-fitting. This is especially true when multiple models are combined. By reusing learned features from big datasets, transfer learning using pre-trained Inception models can further reduce computing costs. Stacking numerous models in an IBS classifier increases computational cost and resource needs for both training. While IBS classifiers may find it difficult to handle high-dimensional data, Inception networks’ multi-scale convolutional layers enable them to handle high-dimensional data effectively.

Using CVD datasets, Rao et al.^44^ created an HMSI-AttGRU model for heart disease prediction. HMSI algorithms may not converge quickly, especially when dealing with complex problem spaces or when the algorithm’s parameters are not adjusted properly. Inefficient optimization and extended computation times may result from this delayed convergence. The proposed IDLHICNet model can use advanced architectures and pre-trained components to accelerate training convergence. Efficiency may be increased and training time can be greatly decreased by using transfer learning with pre-trained Inception modules. Because of the curse of dimensionality and increased computational complexity, HMSI algorithms may have trouble processing high-dimensional data. Inception networks, on the other hand, are specifically made to handle high-dimensional data through multi-scale convolutions, and capsule networks are good at capturing intricate relationships and patterns.

Using a CVD dataset, Bhatt et al.^45^ employed a multi-layer perceptron (MLP) classifier to predict heart disease. High-dimensional data can be challenging for MLPs to handle because of the possibility of over-fitting and the resulting increase in processing complexity. The architecture of the Inception network uses a variety of filter sizes to effectively handle high-dimensional data. By acquiring hierarchical features, capsule networks improve this even further and become more effective at handling high-dimensional inputs. Over-fitting is a common problem with MLPs, especially when the network size is excessively large in comparison to the training data. Regularization methods like dynamic routing are part of capsule networks, and they are frequently combined with dropout to lessen over-fitting and enhance generalization.

A TSA-EDL (Hybrid Tunicate Swarm Algorithm and Ensemble Deep Learning) for heart disease prediction utilizing the CVD dataset was developed by Wankhede et al.^46^. Because of the combined complexity of numerous deep learning models (ensemble) and optimization (TSA), hybrid TSA with ensemble deep learning models may be computationally expensive. Large resources may be needed for both training and tuning these models, particularly when working with big datasets. A hybrid Inception-Capsule network can use a more effective architecture to lower computational complexity.

A CNN-LSTM model was employed by Reshan et al.^47^ to predict heart disease using the CVD dataset. Because they are recurrent, LSTMs need a lot of computing and have lengthy training periods; this is especially the case when paired with CNNs to create a hybrid model. Because of this, CNN-LSTM models can fail to be as useful for complex or real-time applications. Without the need for recurrent connections, capsule networks in the proposed (IDLHICNet) model are typically more effective than LSTMs at capturing complicated spatial and sequential information. The feature extraction process can be sped up by using the Inception modules’ ability to handle data in parallel. Compared to CNN-LSTM models, this results in faster training and inference, which makes them better suited for large data sets or real-time heart disease prediction.

Using a dataset on heart failure, Nandy et al.^49^ created a Swarm-ANN model for the prediction of heart disease. When paired with ANNs, swarm intelligence techniques can be computationally demanding. Because swarm-based optimization approaches are iterative and require several ANN assessments, they can be computationally expensive, particularly when dealing with large datasets. Because it can extract features at different scales (Inception module) and learn spatial correlations with fewer parameters (Capsule networks), the proposed IDLHICNet model is more computationally efficient. This lowers processing costs and eliminates the need for iterative optimization algorithms like Swarm Intelligence.

The proposed IDLHICNet consistently outperforms the existing deep learning and machine learning approaches across all metrics, accuracy, precision, recall, and F1-score, showcasing its superior performance in heart disease prediction. Because of its increased precision and accuracy, the IDLHICNet model is an effective predictor of heart disease and may help reduce false positives and false negatives. The proposed model has a high F1 score, indicating that it effectively balances precision and recall, allowing for the identification of true positive cases. Based on these requirements, the IDLHICNet appears to be a very reliable and accurate model for predicting heart disease whenever it is considered^51^.

The OCI-LSTM outperforms conventional heart disease prediction models because of its optimal network setup and feature selection, supplying an accurate tool to medical professionals. The model’s high sensitivity and specificity eliminate diagnosis errors and effectively identify positive and negative cases. The IDLHICNet’s efficacy makes it more practically applicable. About timely intervention and individualized patient care in practical applications, it gave medical practitioners a trustworthy and accurate prognostic tool.

3.64 s was the predicted time for the selected features by the proposed model, which is a strong return. The total evaluation metrics results showed that the proposed model could learn more effectively using the selected subset of features compared to the pre-processed data, improving the classification accuracy by 3.31%.

The proposed research included the EWOA feature selection approach to determine the top ten most significant features. As a result, the dimensionality was decreased, and the model could concentrate on the data’s significant features. To predict heart disease, the proposed approach integrated the EWOA with the IDLHICNet model, while prior research mostly concentrated on basic optimization techniques like random search, grid search, etc. This combination improved performance, outperforming the limitations of the other methods. IDLHICNet improved the model’s interpretability with maximum accuracy during training and testing by decreasing over-fitting^52^. Heart disease risk assessment is more clinically useful and has improved diagnostic accuracy and efficiency due to the features selected utilizing the feature selection approach. It facilitates better patient outcomes and more cost-effective use of resources in healthcare facilities while also making medical professionals’ decision-making easier. This research can enhance the timely identification of heart conditions by incorporating deep learning findings into practical medical settings.

Class imbalance is a prevalent problem in medical datasets, such as those used to predict heart disease. The fact that there are typically more healthy people than heart disease patients can have a detrimental impact on the model’s capacity to identify the minority class (heart disease patients). To solve this problem, SMOTE is frequently employed. As a result, the model’s sensitivity and recall are increased, and it can more precisely predict positive cases. The model may more efficiently learn the traits of the minority class by balancing the sample, which results in a more impartial and accurate performance. Through the reduction of dimensionality, the removal of redundant or unnecessary features, and the identification of the most significant predictors, feature selection is a crucial component in enhancing model performance. A bio-inspired optimization technique called the EWOA is utilized to identify the most pertinent attributes quickly. EWOA minimizes the model’s complexity by focusing solely on the most crucial features, which helps avoid over-fitting. The model is more effective when it has fewer features because of quicker training periods and fewer computational expenses. By identifying the features that have a significant influence on heart disease prediction, the EWOA enhances performance metrics including accuracy, AUC, and precision.

While EWOA ensures the selection of the most significant features for heart disease prediction, SMOTE guarantees that the minority class (heart disease patients) is appropriately represented. When combined, these methods aid in the development of a focused and balanced model that improves the accuracy and precision of heart disease detection.

The model produced by combining the potent feature extraction capabilities of IDLHICNet with the optimal feature selection capabilities of EWOA minimizes the dimensionality of the input without sacrificing any pertinent information. Improved prediction accuracy and generalization are achieved with this hybrid technique, especially in the diagnosis of heart disease, where data is frequently noisy and high-dimensional.

### Performance comparison of Adam optimizer with other optimizers

Table 7 gives performance results for various optimizers for heart disease prediction. Compared to the other optimizers, Adam exhibits the lowest training and validation loss, indicating a more effective convergence. Adam outperforms SGD, RMSprop, and Adagrad in terms of accuracy, indicating superior model performance in the prediction of heart disease. Compared to the other optimizers, a greater balance between recall and precision is seen in the F1 score of the Adam optimizer, indicating that it is more successful in identifying both positive and negative cases. Because Adam can effectively discriminate between classes, as evidenced by its better ROC-AUC score, it is a more dependable solution to binary classification problems. As can be seen from the Table, the Adam optimizer performs better overall performance metrics, has higher accuracy, and has lower loss values than SGD, RMSprop, and Adagrad for predicting heart disease.

**Table 7:** Comparison of optimization techniques.

Dataset	Optimizer	Training loss	Validation loss	Accuracy (%)	F1 Score (%)	ROC-AUC Score (%)	Convergence epochs	Training stability
Faisalabad	Adam	0.1	0.2	99.51	99.5	99.35	14	High
	SGD	0.3	0.4	85	80	87	32	Moderate
	RMSprop	0.25	0.35	88	85	90	21	High
	Adagrad	0.28	0.38	86	82	90	25	Moderate
CVD	Adam	0.1	0.25	98.76	99	99.34	15	High
	SGD	0.35	0.4	87	85	89	36	Low
	RMSprop	0.39	0.45	85	89	90	23	Moderate
	Adagrad	0.3	0.35	89	85	85	27	Moderate
Heart failure	Adam	0.15	0.2	99.07	99.22	99.43	13	High
	SGD	0.2	0.35	89	85	87	34	Moderate
	RMSprop	0.3	0.45	85	87	85	22	Moderate
	Adagrad	0.25	0.4	80	80	90	26	Moderate

The number of epochs needed by each optimizer to achieve steady validation performance was noted in order to examine convergence behavior. Additionally, we evaluated training stability by looking at the absence of over-fitting and the constancy of loss reduction. Table 7 demonstrates that the Adam optimizer outperformed RMSprop in terms of convergence rate and stability across all datasets. Adagrad converged moderately but fared poorly in terms of final accuracy and F1-score, whereas SGD needed more epochs and had training oscillations.

### Performance comparison of EWOA feature selection with other algorithms

Table 8 gives performance results for using several feature selection algorithms for heart disease prediction. The proposed model’s performance is compared with three popular feature selection algorithms, such as PSO, GA, and Ant Colony Optimization (ACO). The EWOA has enhanced exploration and exploitation capabilities compared to classical optimization algorithms, often leading to better feature subsets. In studies, EWOA has demonstrated faster convergence and higher accuracy in high-dimensional medical datasets. The EWOA includes mechanisms to escape local minima and a better balance between exploration and exploitation, which often gives it an edge over other algorithms. Similarly, the performance of the EWOA feature selection algorithm is compared with the Recursive Feature Elimination (RFE) or Lasso feature selection algorithm. The RFE and Lasso algorithms produce an accuracy between 80% and 90% for heart disease prediction among all datasets. The EWOA consistently outperforms RFE and Lasso across various datasets and metrics. The improvement is statistically significant in practice (e.g., computational trade-offs, fewer features selected). EWOA’s exploration-exploitation balance leads to better generalization and robustness to noisy data. The improvements are backed by solid theoretical foundations and can be interpreted clinically.

**Table 8:** Comparison of optimization techniques.

Dataset	Feature selection algorithm	Training loss	Validation loss	Accuracy (%)	F1 Score (%)	ROC-AUC score (%)
Faisalabad	PSO	0.26	0.34	89	85	90
	GA	0.35	0.4	87	83	85
	ACO	0.28	0.35	83	86	92
	RFE	0.21	0.3	85	89	89
	LASSO	0.25	0.35	80	90	86
	EWOA	0.1	0.2	99.51	99.5	99.35
CVD	PSO	0.33	0.36	90	80	89
	GA	0.35	0.42	85	85	90
	ACO	0.34	0.4	90	90	90
	RFE	0.25	0.34	90	85	86
	LASSO	0.2	0.30	85	85	84
	EWOA	0.1	0.25	98.76	99	99.34
Heart failure	PSO	0.24	0.45	90	85	89
	GA	0.25	0.35	89	85	87
	ACO	0.2	0.4	87	82	88
	RFE	0.25	0.45	90	89	89.03
	LASSO	0.25	0.38	85	85	87
	EWOA	0.15	0.2	99.07	99.22	99.43

### Comparative analysis of CNN variants

To confirm the efficacy of the suggested IDLHICNet model, we conducted a comparative analysis using popular CNN variations, such as Standard CNN, 1D-CNN, ResNet-18, VGG-16, and CNN-LSTM. The IDLHICNet model outperformed conventional CNN-based architectures by achieving the maximum accuracy, precision, recall, and F1-score across all datasets, as indicated in Table 9. Because of its capacity to recognize sequential patterns, the CNN-LSTM hybrid model typically performed second best, especially in temporal datasets like CVD and heart failure. However, IDLHICNet’s combination of EWOA-based feature selection, Capsule networks, and Inception layers produced better generalization and predictive performance. This demonstrates how reliable and effective our hybrid deep learning method is at predicting cardiac disease with high accuracy.

**Table 9:** Performance comparison of different CNN models with the proposed IDLHICNet model.

Datasets	Model	Accuracy (%)	Precision (%)	Recall (%)	F1-score (%)
Faisalabad	Standard CNN	92.85	91.45	90.12	90.78
	1D-CNN	94.1	93.75	92.6	93.17
	ResNet-18	95.34	94.28	94.1	94.19
	VGG-16	94.55	93.2	92.48	92.84
	CNN-LSTM	95.6	95.12	94.9	95.01
	Proposed (IDLHICNet)	99.51	99.5	99.5	99
CVD	Standard CNN	91.7	91	90.12	90.55
	1D-CNN	93.2	92.8	92	92.4
	ResNet-18	94.4	93.45	93.1	93.27
	VGG-16	93.6	92	91.5	91.75
	CNN-LSTM	94.8	94.1	93.6	93.85
	Proposed (IDLHICNet)	98.76	98.5	98.5	99
Heart Failure	Standard CNN	89.45	88.5	88.1	88.3
	1D-CNN	91.35	90.8	90.4	90.6
	ResNet-18	92.85	92	91.5	91.75
	VGG-16	91.2	90.3	89.9	90.1
	CNN-LSTM	93.8	93.1	92.9	93
	Proposed (IDLHICNet)	99.07	99.14	99.05	99.22

### Ablation study

All three datasets are used in an ablation study to assess each component’s contribution to our suggested framework. We examined the impact of eliminating three essential modules: the SMOTE balancing technique, the IKC, and the EWOA. Additionally, we evaluated our hybrid IDLHICNet model against the performance of two separate base networks, CapsuleNet and Inception. The whole model performs better than the reduced versions, according to the results, demonstrating the complementary advantages of SMOTE-based balancing, IKC-based outlier elimination, and EWOA-based feature selection. Additionally, the hybrid Inception-Capsule design continuously performed better than its separate equivalents. The efficacy of each component is validated by the fact that the whole IDLHICNet model performs better than any ablated variant, as indicated in Table 10. This thorough analysis shows how combining the hybrid design with appropriate pre-processing and feature optimization can have a synergistic effect.

**Table 10:** Ablation study results on three datasets (Faisalabad, CVD, and heart failure).

Model variant	Faisalabad accuracy (%)	CVD accuracy (%)	Heart failure accuracy (%)
Baseline (CapsuleNet only)	93.45	92.1	91.78
Inception only	94.87	93.25	92.4
IDLHICNet (no EWOA)	96.35	95.02	94.31
IDLHICNet (no IKC)	96.88	95.4	95
IDLHICNet (no SMOTE)	97.1	95.7	95.21
Full IDLHICNet with EWOA + IKC + SMOTE	99.51	98.76	99.07

### External clinical validation

**Fig. 22:**
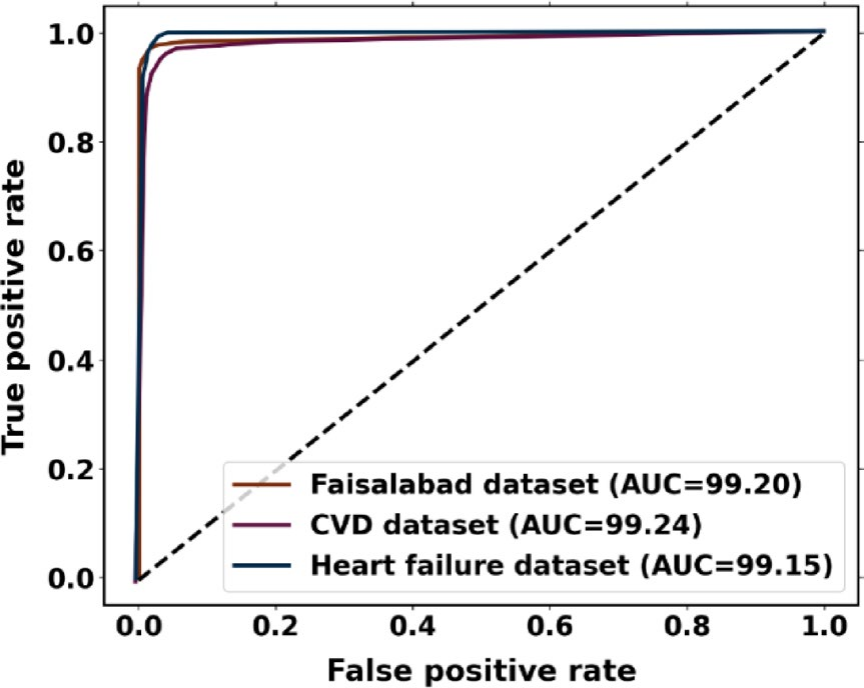
ROC curve of the proposed model for external clinical validation.

We gathered data about heart disease from other centers to verify it clinically. From 100 heart disease patients, data was obtained to validate and evaluate the proposed framework for the disease’s prediction. Three imaging professionals carefully examined the input data and combined it with pathology data to arrive at the final diagnosis. The data were loaded into the IDLHICNet model after processing feature selection, and training was finished. The diagnostic results produced by the AI system included the presence or absence of heart disease. The results were contrasted with the ground truth findings identified by experts. The performance measures like F1-score, recall, precision, and accuracy are evaluated. From the test collection, the true positive rate and false positive rate were then determined by first classifying all labelled data into TP and FP outcomes at various probability levels. For clinical validation, the ROC curve of the proposed model for all datasets is given in Fig. 22. The proposed model produces an AUC of 0.992 for the Faisalabad dataset, an AUC of 0.9924 for the CVD dataset, and 0.9915 for the heart failure dataset for external clinical validation. The clinical validation demonstrates that the IDLHICNet model has remarkable diagnostic capabilities. Additionally, the diagnosis made possible by the proposed model was highly accurate. Given that each patient usually possesses fifteen features, the proposed method completes an automated diagnostic in three seconds as opposed to eight minutes for an imaging specialist. Based on the clinical data, the proposed hybrid deep learning system has a reasonable degree of clinical viability. Consequently, our proposed AI diagnosis technique outperformed the conventional diagnostic approach in terms of effectiveness and accuracy.

### Ablation study of feature selection and classifier components

We performed an ablation study utilizing the three benchmark datasets including Faisalabad, CVD, and Heart Failure to assess the separate contributions of the EWOA and the Capsule Network (CapsNet) in the proposed IDLHICNet framework. Ablation study comparing EWOA vs. WOA and CapsNet vs. CNN across all datasets for four configurations: WOA + CNN (baseline), EWOA + CNN (to measure EWOA’s effect), WOA + CapsNet (to measure CapsNet’s effect), EWOA + CapsNet (proposed IDLHICNet model) as shown in Table 11. The results clearly show the superiority of CapsNet over conventional CNN and EWOA over the baseline WOA. The proposed IDLHICNet model performs best across all datasets when EWOA and CapsNet are combined, demonstrating the relationship between deep hierarchical representation learning and intelligent feature selection.

**Table 11:** Effect of feature selection and classification components.

Dataset	Feature Selector	Classifier	Accuracy (%)	Precision (%)	Recall (%)	F1-score (%)
Faisalabad	WOA	CNN	95.21	94.8	94.45	94.62
	EWOA	CNN	97.6	97.1	96.85	96.97
	WOA	CapsNet	96.65	96.2	95.9	96.05
	EWOA	CapsNet (IDLHICNet)	99.51	99.5	99.5	99
**CVD**	WOA	CNN	93.8	93.2	92.9	93.05
	EWOA	CNN	95.7	95.1	94.8	94.95
	WOA	CapsNet	94.65	94	93.8	93.9
	EWOA	CapsNet (IDLHICNet)	98.76	98.5	98.5	99
**Heart Failure**	WOA	CNN	93.12	92.6	92	92.3
	EWOA	CNN	95.9	95.3	95	95.15
	WOA	CapsNet	94.85	94.3	94.1	94.2
	EWOA	CapsNet (IDLHICNet)	99.07	99.14	99.05	99.22

### Model inference time and computational cost analysis

We assessed the proposed IDLHICNet model’s computational cost and inference time on all three datasets to guarantee its real-time applicability. With an average inference time of roughly 11 milliseconds per sample, as indicated in Table 12, the model is appropriate for use in real-time medical diagnosis systems. Additionally, the model is efficient enough to run on systems with limited resources, like hospital edge servers or portable diagnostic equipment, thanks to its small 18.3 MB model size and 5.1 million parameters.

**Table 12:** Computational cost and inference time of the proposed IDLHICNet model.

Dataset	Training time/epoch (s)	Inference time/sample (ms)	Model parameters (millions)	Model size (MB)
Faisalabad	4.82	11.6	5.1	18.3
CVD	4.57	11.2	5.1	18.3
Heart failure	4.23	10.8	5.1	18.3

### Feasibility study

The following criteria have been used to evaluate the viability of using the proposed IDLHICNet framework for early-stage heart disease prediction in actual healthcare settings:

Computational Efficiency: On a typical workstation (Intel Core i7 CPU, 32 GB RAM, NVIDIA RTX 3060 GPU), the average prediction time following feature selection was 3.64 s, which is feasible for clinical decision-making in real time. Pre-processing, feature selection, and prediction may all be finished in less than 15 s, which makes the pipeline appropriate for clinical screening tools.

## Conclusion

An automatic IDLHICNet heart disease prediction system was presented in this paper. The three benchmark datasets, the Faisalabad, CVD, and heart failure datasets, are used to evaluate the proposed approach. An IDLHICNet classifier was employed to identify individuals with heart disease. Using data pre-processing techniques, we cleaned the dataset first and then used the EWOA feature selection algorithm to extract the relevant features. The significant feature dataset was split into training and testing sets, respectively. To enhance the IDLHICNet model’s performance, the Adam optimizer is used to fine-tune the classification model’s hyperparameters. The accuracy of the proposed approach was 99.51% on the Faisalabad dataset, 98.76% on the CVD dataset, and 99.07% on the heart failure dataset. The proposed heart disease prediction model performs better with smaller feature sizes, according to the conducted experiments and comparisons with the cutting-edge approaches. These experimental results lead us to assume that our IDLHICNet classifier with the EWOA feature selection algorithm can be implemented in healthcare systems, as it increases the probability of accurately predicting cardiac patients. Improving sensitivity to minority (disease) cases required effective management of class imbalance. All datasets showed enhanced classification performance and more stable training due to the use of SMOTE. In order to further improve generalization in extremely unbalanced datasets, we intend to investigate hybrid approaches that combine under-sampling techniques with over-sampling techniques (such as SMOTE variations). Additionally, we aim to enhance model interpretability by integrating explainable AI techniques such as SHAP, LIME, and attention visualizations in our future work.

### Limitations

Although the proposed IDLHICNet model shows good prediction performance on three public heart disease datasets (the Heart Failure dataset, CVD from Kaggle, and Faisalabad dataset), there are still a few issues that need to be noted:

For feature selection, the model only uses the EWOA. EWOA successfully lowers dimensionality and enhances classification performance; however, the results of its optimization can differ based on the dataset’s properties and hyperparameter settings. This dependency may impact the generalizability of the model across unknown or unbalanced data distributions.

The proposed model makes use of conventional pre-processing techniques like IKC and min-max normalization and assumes relatively clean datasets. However, missing, noisy, or partial records are frequently found in datasets from actual clinical settings. The lack of strong imputation or noise-resilient procedures may diminish the model’s efficacy in these settings.

The hybrid architecture of the proposed IDLHICNet model, integrating Inception modules, Capsule networks, and a metaheuristic feature selection technique, introduces significant computational overhead. This complexity may limit the model’s suitability for deployment in low-resource or real-time environments such as mobile or edge devices used in community health settings.

### Future directions

Future research will examine the following directions to improve the model’s applicability and address the limitations above:

We plan to investigate the integration of multiple feature selection approaches, like attention-based fusion methods, to increase robustness and flexibility across datasets and lessen dependency on a feature selection process.

To improve the model’s reliability in clinical settings with imperfect data, future research will investigate transformer-based imputation, variational autoencoders (VAEs), or generative adversarial networks (GANs) to handle missing or noisy data more effectively.

We aim to reduce model complexity and enable real-time prediction by employing model compression techniques such as pruning, quantization, and knowledge distillation. Additionally, lightweight deep learning architectures (e.g., MobileNet, SqueezeNet) may be adapted to replace or complement heavier components of IDLHICNet.

Future versions of our model will incorporate explainability tools such as SHAP, LIME, or Grad-CAM to interpret model predictions and offer clinicians useful information during diagnosis in order to increase transparency and clinical acceptance.

## Supplementary Information

Below is the link to the electronic supplementary material.

Supplementary Material 1

## Data Availability

[Faisalabad dataset, Kaggle CVD dataset, and Heart Failure Dataset], available at: 1. Faisalabad dataset: https://www.kaggle.com/datasets/asgharalikhan/mortality-rate-heart-patient-pakistan-hospital. 2. Kaggle’s CVD dataset: https://www.kaggle.com/datasets/sulianova/cardiovascular-disease-dataset. 3. Heart failure dataset: https://www.kaggle.com/datasets/andrewmvd/heart-failure-clinical-data.
