# Supplementary material for: Improved Inception-Capsule deep learning model with enhanced feature selection for early prediction of heart disease
Source: Sci Rep. 2025 Sep 25;15:32847. doi: 10.1038/s41598-025-18551-4 (PMC12464333; doi:10.1038/s41598-025-18551-4)
Supplement: Supplementary file 1 — Supplementary Material 1 [file 41598_2025_18551_MOESM1_ESM.docx]

**Using chaotic reverse elite people as the basis for population initialization:**

The whale group's starting location in the whale optimization method is heavily constrained by prey [42]. A more substantial difference between the whale group and the target allows the whale to feed on it more quickly. As a result, determining the starting whale population's position is crucial to determining the algorithm's suitable value. Various meta-heuristic algorithms use the chaos method. The initialization populations are generated using elite opposition-based learning because it is an effective improvement technique for population initialization. Nevertheless, developing the algorithm's initial population only through elite opposition-based learning or the chaotic technique ignores the retention of elite people and the initial population's uniform distribution in the solution space. Therefore, by fusing the Elite Opposition-Based Learning approach with the Gaussian chaos technique based on the chaotic reverse elite individuals, this research provided an initialization technique for the population.

The probability that the method will identify the optimal number increases with the initial population's uniform distribution throughout the solution space. Because of its non-repetition, ergodicity, randomness, and other qualities, chaotic search is more frequently utilized in creating initial populations than a random-search approach. Nonetheless, distinct chaotic maps impact the algorithm's starting population differently. For the whale optimization method, the appropriate chaotic map was chosen in this research using the examination and contrast of the Chebyshev, Tent, Gauss, and Rand maps.

Using chaotic maps, specifically Gauss, Chebyshev, and Tent functions, the initial whale population was created to enhance diversity. Using the Gauss map, the whale population made from the initial whale population production perspective is more uniformly dispersed in space, offering a more robust assurance for the algorithm's global optimization.

Gauss/mouse map:

$q_{k+1}=\left\{ \begin{matrix} 1, & q_{k}=0 \\ \frac{1}{mod \left( q_{k},1 \right)}, & otherwise \end{matrix} \right\}$ (1)

Where the $k^{th}$ chaos number is $q_{k}$, the number of iterations is represented as *k*, $q\in\left( 0,1 \right)$.

Based on the Elite Opposition-Based Learning and Gauss/mouse map, the chaotic initialization strategy reverses the elite individual population indicated in this research.

**Probability selection strategy based on the skew distribution:**

WOA implies that there is a 50% chance that a group of whales would decide to surround and feed, and that each algorithm iteration's probability p will follow a uniformly distributed random number between [0,1], which deviates from real-world animal hunting guidelines. In the natural world, a predator's probability of approaching and consuming its prey varies over time [37]. There is no uniform distribution for the probability of its generation.

A new probability generation method is presented to improve WOA's capacity for global exploration. Three components make up this method's partition of the WOA iteration process, and the probabilities generated during each interval are subsequently rectified.

1. Early iteration: Through the position-sharing system, the remaining whales swiftly migrate near the suitable individual when they discover prey. There is currently a greater than 0.5 chance of surrounding the prey. As a result, the early algorithm iteration's probability of hunting behavior has a distribution with a negative skew of 0.8.

2. Mid iteration. The prey has already been surrounded by the whales in close proximity, while distant groups continually approach the prey. Every whale's probability of surrounding and feeding at this moment is the same. Consequently, the chance of the whale encircling and hunting during this time follows the uniform distribution in the interval $\left[0,1\right]$.

3. Late iteration. I considered that the whale group had encircled the prey and was now being attacked. However, because the prey is motivated to survive, the whale's probability of successfully preying on it is less than 0.5. As a result, a 0.2 positive skew distribution describes the whale enclosure and hunting behavior probability of the late algorithm iteration.

The equation for generating probability is provided as follows by the description above:

(2)

Where the proposed approach for generating random numbers with a skew distribution is $rsn\left( n,location,scale,shape \right)$, and the algorithm's maximum iterations are represented as $t_{max}$.

Equation (3) shows that the produced probability range is not in the interval $\left[0,1\right]$. As a result, the established probability's boundary is constrained, namely.

$p=\left\{ \begin{matrix} 1, & p>1 \\ 0, & p<1 \end{matrix} \right\}$ (3)

**Non-linear correction strategy of a and $\overset{\boldsymbol{\to}}{\mathbf{C}}$:**

Under the guidance of two crucial WOA parameters, such as $\overset{\to}{A}$ and $\overset{\to}{C}$, whales can search, surround, and prey on prey. The factor of convergence determines the value of $\overset{\to}{A}$. In the convergence factor, the linear lower trend observed implies a decreasing trend in the prey's distance from the whale. The distance between the whale and its prey is said to vary randomly when $\overset{\to}{C}$an evenly distributed random value between 0 and 2 is. The algorithm's local mining and worldwide exploration, $\overset{\to}{C}$ have no discernible impact.

Consequently, the values of a and $\overset{\to}{C}$ are modified to increase the WOA's convergence rate, which is described as,

(4)

$\overset{\to}{C}=2rsn\left( 1,1,0.7,0.1 \right)$ (5)

Where $a\in\left[ 2,0 \right]$,$t_{max}$ is the algorithm’s maximum iterations are described as $t_{max}$, and the current number of iterations is described as *t*.

**Location update strategy:**

When $\left| \overset{\to}{A} \right|<1$, the WOA whale group surrounds a suitable whale individual and moves towards the prey. A non-linear decreasing disturbance component is added to the algorithm to boost its local mining capability and convergence accuracy, hence hastening the process by which whales go to the best individual whale and rapidly surround their target.

$\overset{\to}{Q_{*}}\left( t \right)-\overset{\to}{A}\times\overset{\to}{D}\times\omega$, $\left| \overset{\to}{A} \right|<1$ (6)

In the current whale population, the optimal solution's position vector is represented by $\overset{\to}{Q_{*}}\left( t \right)$, *ω* is used to describe the non-linear decreasing perturbation factor. A description of a coefficient vector is $\overset{\to}{C}$.

$\omega=0.8\times\cos\left( \frac{\pi}{2t_{max}}() \right)$ (7)

The revised equation for the position update is:

$\left\{ \begin{matrix} \left\{ \begin{matrix} \overset{\to}{Q_{*}}\left( t \right)-\overset{\to}{A\times}\overset{\to}{C}\times\omega, & \left| \overset{\to}{A} \right|<1 \\ \left( \overset{\to}{Q_{*}}\left( t \right)-\overset{\to}{A\times}\overset{\to}{C} \right), & \left| \overset{\to}{A} \right|\geq1 \end{matrix}, \right. & p<0.5 \\ \overset{\to}{D}\times e^{bl}\times\cos\left( 2\pi l \right)+\overset{\to}{Q_{*}}\left( t \right), & p\geq0.5 \end{matrix} \right\}$ (8)

The probability produced by Equations (2) and (3) is denoted by p, and the distance between other individuals and the optimal whale individual is described as $\overset{\to}{D}=\overset{\to}{C}\times\overset{\to}{Q_{*}}\left( t \right)-\overset{\to}{Q}\left( t \right)$.

**Classification**

**Inception ResNet layer:**

Essential modules are beneficial in creating a very detailed and comprehensive model. The Inception modules perform convolutions with varying kernel sizes concurrently and then connect the outcomes of these parallel computations. This block's input comes from the layer before it. It relates to Maxpool 1D, a $1*3$ max pooling technique, and all four convolutional layers with $1*1$ dimension $(Conv1D)$. This low-cost $1*1 Conv1D$ option keeps the relevance of the input features while reducing their dimensionality. Eliminating the excess channel is a far more cost-effective process. The input channels are reduced to one by this $1*1 Conv1D$.

The input functions for three more convolution layers in Inception A ($1*1$ and two$1*3$). Next, the reducing layer receives the output. It is then subjected to Inception B and Inception C. In Fig. 3, details of each of these blocks are provided. The following applies a dropout with a 0.80 drop rate and average pooling. Next, a feature selection layer is created using the dropout output, which is provided to the capsule layer in vector form at the end of this process.

The mathematical expression for feature vectors for all datasets is given in equations (9), (10) and (11): For the Faisalabad dataset, $n=12$, so each feature vector has 12 components,

$x_{i}^{Faisalabad}=\left[ x_{i1},x_{i2},...,x_{i12} \right]$ (9)

For the CVD dataset, $n=12$, so each feature vector has 12 components:

$x_{i}^{kaggle}=\left[ x_{i1},x_{i2},...,x_{i12} \right]$ (10)

For the heart failure dataset, $n=30$, so each feature vector has 30 components:

$x_{i}^{heartfailure}=\left[ x_{i1},x_{i2},...,x_{i30} \right]$ (11)

**Capsule layer (Caps):**

The Capsule layer receives features encoded with ResNet. A capsule is a collection of neurons whose activity vectors provide the sampling parameters; the vector's length indicates the probability of a feature's presence. This network comprises the class capsule layer, primary capsule layer (PC), and a convolutional layer. After the first capsule layer, several capsule layers are formed, which are known as the class capsule layer. The final capsule layer is the last. The convolution layer extracts the features of the data and forwards them to the first capsule layer. An activity vector $u_{i}\in R$ is present in each capsule *i* (where $1 \leq i\leq N$) in layer l to encode spatial data as sample parameters. All capsules in the next layer 𝑙 + 1 receive the output vector $u_{i}$ from the lower-level capsule 𝑖. When layer 𝑙 + 1's $j^{th}$ capsule receives $u_{i}$, it discovers its product with the matching weight matrix $w_{ij}$.

There are some capsules in this layer. To capture the input features, the caps create vector-valued capsules from the scalar features that the Inception ResNet layer gathered. The prediction vector *M* may be derived using the following equation, given a weighted matrix $w_{ij}$ and an InceptionResNet output of $h_{i}$:

${\overset{\wedge}{t}}_{i\backslash j}=w_{ij}h_{i}$ (12)

Each prediction vector $\overset{\wedge}{t_{i|j}}$'s weighting set, which is determined by the following equation, makes up the set of inputs to a capsule $z_{i}$:

$Z_{i}=\sum_{i=1}^{N} c_{ij}.{\overset{\wedge}{t}}_{i|j}$ (13)

Where the coupling coefficient is described as $c_{ij}$, which the Dynamic Routing algorithm continuously modifies. The values of k vectors are mapped to [0-1] using the "squash" as a non-linear function. The following equation is used to apply this function to $Z_{j}$:

$v_{j}=\frac{\left\| z_{j} \right\|^{2}z_{j}}{1+\left\| z_{j} \right\|^{2}\left\| z_{j} \right\|}$ (14)

A capsule's output is a vector; it may be given to a higher-level capsule that has been chosen. Dynamic Routing was employed as the routing mechanism in the proposed architecture.

**Self-attention layer:**

To enhance machine translation efficiency, specifically, an encoder-decoder model attention mechanism was developed. The attention method enabled the decoder to flexibly utilize the most relevant segments of the input sequence by using a weighted composite of all encoded input vectors, with the most pertinent vectors receiving the largest weights. The goal of data mining is to focus on the most important features of the data. The mapping of attention is:

$Attention\left( q,K,V \right)=\sum_{i=1}^{K} softmatch_{a}\left( q,k \right)_{i}.v_{i}$ (15)

Where $q\in Q$ a query, $Q\subseteq R_{q}^{d}$ the query space, $K\subseteq R_{k}^{d}$ the key space and $K=\left\{ k_{1},...,k_{N} \right\}\subseteq K$, $V\subseteq R_{v}^{d}$ the value-space and $V=\left\{ v_{1},...,v_{N} \right\}\subseteq V$, $softmatch_{a}\left( q,k \right)_{i}$ is a probability distribution over the elements of 𝐾 defined as:

$softmatch_{a}\left( q,k \right)_{i}=\frac{\exp\left( a\left( q,k_{i} \right) \right)}{\sum_{j=1}^{N} \exp\left( a\left( q,k_{i} \right) \right)}=soft\max\left( a\left( q,k_{i} \right) \right)$ (16)

Moreover, when $K=V=Q,$ self-attention can be defined as:

$Q\mapsto SelfAttention\left( Q \right):=Attention\left( Q,Q,Q \right)$ (17)

**Classification Layer:**

In the 𝑛 neurons' fully connected layer (n=number of classes), the self-attention layer's flattened outputs (*F*) are sent

$P=W_{dense}*F$ (18)

The probability of each member of the 𝑛 class should be represented by the output of 𝑃. The Softmax function, computed for each $f_{i}\subseteq F$, is utilized for this purpose:

$P_{i}=\frac{e^{-f_{i}}}{\sum_{f} {{}_{j\in f}e}^{-f_{i}}}$ (19)

**Cross entropy loss function**

It is frequently utilized in deep learning models for data classification. It is also commonly employed in multiple logistic regression. Typically, the gradient descent algorithmic network structure serves as the foundation for the cross-entropy loss function. While producing issues like slower gradient updates, adding cross-entropy loss functions to neural networks helps counteract the saturation tendencies caused by the (inverse) Softmax function. Similarly, gradient descent computations using a cross-entropy loss function might prevent gradient dispersion but may result in decreasing learning rates. As a result, the cross-entropy loss function; the labels given to the actual samples are$\left[0,1\right]$, which stand for the positive and negative situations, respectively. In this research, a sample with perfect attributes is denoted by a 1, while samples with imperfections are denoted by a 0. The model generates a probability value using the sigmoid function.
